# Comparative Evaluation of Feature Selection Strategies for ICU Mortality Prediction Using Chest Radiographic, Radiomic, and Demographic Features

**DOI:** 10.3390/diagnostics16152391

**Published:** 2026-07-30

**Authors:** Orhan Gok, Türker Fedai Çavuş, Omer Ozdemir, Ahmed Cihad Genç, Selcuk Yaylacı, Laçin Tatlı Ayhan

**Affiliations:** 1 Department of Electrical and Electronics Engineering, Faculty of Engineering, Sakarya University, Sakarya 54050, Türkiye; orhan.gok.eem@gmail.com; 2 Department of Electrical and Electronics Engineering, Faculty of Engineering, Sakarya University of Applied Sciences, Sakarya 54187, Türkiye; omerozf@gmail.com; 3 Ahmed Cihad Genc Private Clinic, Istanbul 34480, Türkiye; genccihad@gmail.com; 4 Department of Internal Medicine, Faculty of Medicine, Sakarya University, Sakarya 54100, Türkiye; selcukyaylaci@sakarya.edu.tr; 5 Department of Radiology, Faculty of Medicine, Sakarya University, Sakarya 54100, Türkiye; drlacintatli@gmail.com

**Keywords:** intensive care unit, mortality prediction, chest radiography, machine learning, feature selection, radiomics, MRMR, clinical decision support

## Abstract

**Background/Objectives:** This study investigated the impact of feature type, feature dimensionality, and feature selection strategies on intensive care unit (ICU) mortality prediction using first-day portable frontal chest radiographs and demographic data. In addition, the study evaluated whether comparable predictive performance could be achieved using reduced, clinically interpretable feature sets. **Methods:** A total of 500 patients were included, comprising 400 cases for model development and 100 independent cases for testing. Two complementary experimental frameworks were evaluated. In Experiment Set-1, a clinically interpretable feature set consisting of 12 radiographic and demographic variables was analyzed. In Experiment Set-2, a high-dimensional feature space consisting of 74 radiographic, radiological, radiomic, and image-derived features was investigated using six feature selection methods: ANOVA, Chi-Square, Kruskal–Wallis, MRMR, ReliefF, and Shapley-based importance analysis. Machine learning models were evaluated using the area under the receiver operating characteristic curve (AUC), sensitivity, specificity, accuracy, and F1-score. **Results:** In Experiment Set-1, feature selection reduced the number of predictors from 12 to 4. Using the complete 12-feature set, the Bagged Trees classifier achieved the highest performance (AUC = 0.95). Following feature reduction, the Subspace KNN classifier achieved an AUC of 0.96 with an accuracy of 0.82, indicating that satisfactory predictive performance could be achieved using a substantially smaller feature set. In Experiment Set-2, comparison of six feature selection strategies demonstrated that MRMR and Kruskal–Wallis produced the highest-performing feature subsets. Cobb angle, bilateral infiltrates, bilateral pleural effusion, and unilateral pleural effusion were consistently identified across all feature selection methods as stable predictors associated with ICU mortality. These predictors were considered stable because they were consistently identified across multiple independent feature selection methods despite differences in the underlying selection algorithms. **Conclusions:** The findings suggest that appropriate feature selection strategies may reduce model complexity while retaining clinically relevant predictive information. The consistent identification of a small group of radiographic features across multiple feature selection methods in the 74-feature experimental framework suggests that these variables represent robust and clinically meaningful predictors of ICU mortality. Further external validation using larger multicenter datasets is required before clinical implementation.

## 1. Introduction

Mortality rates remain considerably high among critically ill patients admitted to intensive care units (ICUs). Therefore, reliable mortality prediction at an early stage may assist clinicians in resource allocation, risk stratification, treatment planning, and timely intervention for high-risk patients [1,2,3]. In this context, chest radiography, particularly portable frontal chest radiographs, is one of the most frequently used imaging modalities in routine clinical practice and plays an important role in the initial assessment of pulmonary and cardiovascular abnormalities [4,5,6].

In this study, portable frontal chest radiographs obtained within the first 24 h of admission from adult patients hospitalized in an Internal Medicine ICU were analyzed using machine learning techniques. Two complementary feature spaces were investigated. The first consisted of a clinically interpretable feature set including radiographic findings, semi-automatically measured image parameters, and demographic variables. The second consisted of a high-dimensional feature space composed of radiographic, radiological, and image-derived features extracted from chest radiographs [7,8,9]. Through the evaluation of these complementary feature spaces, the study aimed to determine whether reliable mortality prediction could be achieved using a limited number of clinically meaningful features while maintaining predictive performance.

The primary novelty of this study lies in the comparative evaluation of feature selection strategies and feature dimensionality in ICU mortality prediction. Unlike many previous studies that focused primarily on maximizing predictive performance through large numbers of radiomic or clinical variables, the present study systematically investigated how different feature selection methods influence model performance and feature stability [10]. Six feature selection approaches, namely ANOVA, Chi-Square, Kruskal–Wallis, MRMR, ReliefF, and Shapley-based importance analysis, were comparatively evaluated. Furthermore, the consistency of selected features across different feature selection methods was examined to identify a robust consensus feature subset within the high-dimensional (74-feature) experimental framework. The findings provide insight into the relationship between feature reduction, model interpretability, and predictive performance, thereby contributing to the development of more transparent and clinically applicable machine-learning-based decision-support systems [11,12].

Despite the widespread use of chest radiography in intensive care units, it remains unclear which radiographic findings provide the most robust prognostic information for early mortality prediction [13,14,15]. Several routinely assessed radiographic variables, including pulmonary infiltrates, pleural effusions, cardiomegaly, thoracic alignment (Cobb angle), and cardiothoracic ratio (CTR), are biologically plausible indicators of disease severity because they reflect respiratory compromise, cardiovascular burden, fluid overload, and structural thoracic abnormalities [16,17,18]. However, the relative importance and stability of these predictors across different feature selection strategies have not been systematically investigated.

Two complementary experimental frameworks were designed to evaluate different aspects of mortality prediction. Experiment Set-1 investigated whether clinically interpretable radiographic and demographic variables were sufficient for accurate prediction, whereas Experiment Set-2 examined the influence of high-dimensional feature spaces and multiple feature selection strategies on model performance and feature stability.

Accordingly, the present study was designed to investigate how feature type, feature dimensionality, and different feature selection strategies influence ICU mortality prediction while identifying robust and clinically interpretable predictors that remain stable across multiple selection methods.

Unlike our previous study, which primarily focused on developing and evaluating machine learning models for ICU mortality prediction using a high-dimensional radiographic feature set, the present study specifically investigates the influence of feature dimensionality and systematically compares six different feature selection strategies [19,20,21,22]. In addition, the study introduces two complementary experimental frameworks and evaluates feature selection consistency to identify a robust consensus feature subset, thereby emphasizing model interpretability and methodological transparency rather than predictive performance alone.

## 2. Materials and Methods

The primary objective of this study was to investigate how feature type, feature dimensionality, and feature selection strategies influence ICU mortality prediction performance. To address this objective, two complementary feature spaces were evaluated. The first consisted of a clinically interpretable feature set derived from radiographic findings, semi-automatically measured image parameters, and demographic variables. The second consisted of a high-dimensional feature space composed of radiographic, radiological, and image-derived features extracted from chest radiographs [7,12]. Machine learning algorithms were subsequently applied to both feature spaces, and the effects of feature reduction and feature selection strategies on predictive performance were systematically evaluated.

For clarity, radiographic features refer to manually evaluated findings identified by expert radiologists, whereas image-derived features denote automatically extracted intensity, texture, and radiomic descriptors computed from chest radiographs [8,11].

### 2.1. Data Preprocessing

Within the scope of this study, retrospective chest radiographs obtained using a portable X-ray device ( SG Healthcare Co., Ltd., Seoul, Republic of Korea) were analyzed. Initially, 510 portable frontal chest radiographs obtained from adult (≥18 years) non-COVID patients admitted to the Internal Medicine Intensive Care Unit of Sakarya Education and Research Hospital were screened for eligibility. Only patients with a single ICU admission, a first-day portable frontal chest radiograph of sufficient diagnostic quality, and complete demographic information were included. Radiographs with severe image artifacts or insufficient image quality that prevented reliable radiological evaluation were excluded from the study (*n* = 10). Consequently, the final study cohort consisted of 500 patients. All included patients had complete imaging and demographic information for the variables analyzed; therefore, no missing-data imputation procedures were required. All radiographs were obtained using the SG Healthcare Jumong Mobile system (SG Healthcare Co., Ltd., Seoul, Republic of Korea), routinely employed in the hospital, with patients in the supine position and at an imaging distance of approximately one meter [4].

A total of 500 patient radiographs were included in the study. The dataset was divided into 400 training cases and 100 independent test cases. The independent test set was not used during feature selection, algorithm comparison, model development, or parameter optimization and was reserved exclusively for final performance evaluation. This study design ensured an unbiased estimation of model generalizability while preventing information leakage during model development. The primary study outcome was ICU mortality. Patients who died during their ICU stay were classified as non-survivors, whereas those discharged alive were classified as survivors. Mortality prediction was performed using first-day portable frontal chest radiographs. The final cohort of 500 patients was randomly divided in MATLAB Online (R2025b and R2026a; MathWorks, Natick, MA, USA) into a training dataset (*n* = 400) and an independent test dataset (*n* = 100).

No data augmentation techniques were applied, and all analyses were conducted using original radiographic images. The dataset was intentionally maintained in its original form in order to preserve the natural characteristics of the clinical data and to evaluate model performance under realistic conditions. The primary objective of the study was not to maximize classification performance through artificial expansion of the dataset, but rather to investigate the effects of feature type, feature dimensionality, and feature selection strategies on mortality prediction using real-world clinical data.

This approach also enabled a direct comparison of different feature sets and feature selection methods under identical experimental conditions. Consequently, the study provides insight into whether reliable mortality prediction can be achieved using reduced and clinically interpretable feature sets without relying on data augmentation procedures.

The sample size was determined by the availability of consecutive eligible patients meeting the predefined inclusion criteria during the study period. Although no formal a priori power calculation was performed because of the retrospective machine-learning study design, the dataset was considered adequate for comparative evaluation, as all machine learning models and feature selection methods were developed using the same training dataset and evaluated on the same independent test dataset under identical experimental conditions.

### 2.2. Ethics Committee Approval

Written permission for the study was obtained from the Non-Interventional Clinical Research Ethics Committee of Sakarya University Training and Research Hospital, where the study was conducted (decision date: 23 February 2024, decision no: 340184-40).

### 2.3. Feature Extraction

Two complementary feature sets were constructed for mortality prediction. The first feature set consisted of clinically interpretable variables derived from radiographic assessment, semi-automatically measured image parameters, and demographic information. Using a custom-developed Python-based image processing pipeline, two quantitative image-derived features, namely the cardiothoracic ratio (CTR) and Cobb angle, were automatically calculated from chest radiographs [11,16,17,18]. In addition, chest radiographs were independently reviewed by experienced clinicians, and seven radiological findings were manually identified.

The clinically interpretable feature set included cardiomegaly (CTR > 0.50), unilateral infiltration, bilateral infiltration, unilateral pleural effusion, bilateral pleural effusion, pneumothorax, calcified aorta, cardiothoracic ratio (CTR), Cobb angle, age, blood group, and gender, resulting in a total of 12 features. By combining radiographic findings with demographic information, this feature set was designed to provide a clinically meaningful and easily interpretable framework for mortality prediction.

Blood group was included as a candidate predictor because previous studies have reported potential associations between blood groups and disease severity, inflammatory response, and mortality in critically ill patients [23,24,25]. Therefore, it was considered a clinically relevant demographic variable for initial feature evaluation. Accordingly, blood group was retained as a candidate predictor for subsequent feature selection.

To improve the reproducibility of the proposed methodology, additional details regarding the feature extraction procedure are provided. All chest radiographs were converted to PNG format and resized to a uniform resolution of 512 × 512 pixels prior to analysis. No lung segmentation was performed prior to feature extraction because the objective of this study was to evaluate global image-derived characteristics obtained directly from portable frontal chest radiographs under standardized preprocessing conditions [7,8,9]. No additional image intensity normalization or filtering procedures were applied. Texture features, including GLCM- and Haralick-based descriptors, together with the remaining image-derived features, were extracted using a custom-developed Python pipeline that was applied consistently to all radiographs. MATLAB (MathWorks, Natick, MA, USA) was subsequently used for feature selection, machine learning model development, cross-validation, and performance evaluation. The complete list of all 74 extracted features is presented in Supplementary Table S1, whereas the detailed GLCM and Haralick descriptors are provided separately in Supplementary Table S2. The detailed GLCM- and Haralick-based texture descriptors included within this dataset are provided separately in Supplementary Table S2. The dataset contained no missing values; therefore, no data imputation procedure was required. All feature extraction procedures and software settings were applied consistently to every image to ensure reproducibility of the analysis.

In addition to the clinically interpretable feature set, a high-dimensional feature space consisting of 74 radiographic, radiological, and image-derived features was constructed. This feature space included radiological findings, intensity-based descriptors, histogram-based parameters, texture measures derived from gray-level co-occurrence matrices (GLCM), Haralick texture features, and other quantitative image characteristics [11,12]. The high-dimensional feature set was subsequently used to evaluate the effects of multiple feature selection strategies and feature reduction approaches on model performance.

The use of both low-dimensional and high-dimensional feature spaces enabled a comprehensive investigation of the relationship between feature complexity, feature selection, model interpretability, and mortality prediction performance.

All image-derived measurements, including the cardiothoracic ratio (CTR), Cobb angle, and radiomic features, were extracted using the same custom-developed Python software under an identical processing pipeline [9,15]. Since all radiographs were processed using the same standardized methodology, measurement consistency was maintained across the entire dataset.

Radiological image interpretation was performed by a single board-certified radiologist using predefined evaluation criteria. Because image interpretation was performed by a single experienced radiologist, interobserver variability was not assessed and is acknowledged as a limitation of the study. During image assessment, the radiologist was blinded to all patient-related clinical information, including demographic characteristics, laboratory findings, and clinical outcomes, and evaluated only the chest radiographs. In addition to the automatically extracted image-derived features generated using the custom-developed Python software, radiological findings were manually assessed by the radiologist. To enhance the methodological rigor of the study, the overall clinical methodology and medical interpretation were reviewed by two board-certified Internal Medicine specialists. Methodological decisions regarding image processing, feature extraction, and machine learning analyses were reviewed jointly by the multidisciplinary author team until consensus was achieved.

For transparency and reproducibility, a complete list of all 74 extracted features, including their definitions, feature categories, and extraction methods, is provided in Supplementary Table S1.

#### 2.3.1. Cobb Angle Measurement

The Cobb angle is the most widely used parameter for quantifying the severity of spinal curvature and is considered the standard method for scoliosis assessment in clinical practice [9,16]. The measurement is performed by identifying the upper and lower vertebrae that define the limits of the curvature and exhibit the greatest inclination relative to the horizontal plane [16,17]. Lines are drawn parallel to the superior endplate of the upper vertebra and the inferior endplate of the lower vertebra. Perpendicular lines are then constructed from these reference lines, and the angle formed by their intersection is measured as the Cobb angle [9,17].

Although the Cobb angle is conventionally used for scoliosis assessment, in the present study it was considered solely as an automatically extracted quantitative descriptor of thoracic spinal alignment visible on portable frontal chest radiographs [9,16,17].

Although Cobb angle measurement is routinely performed by clinical experts, the procedure is subject to inter-observer variability. Consequently, the use of image-processing techniques and computational algorithms to support Cobb angle estimation has attracted increasing interest, as these approaches may improve measurement objectivity, reproducibility, and consistency [9,11].

**Figure 1:**
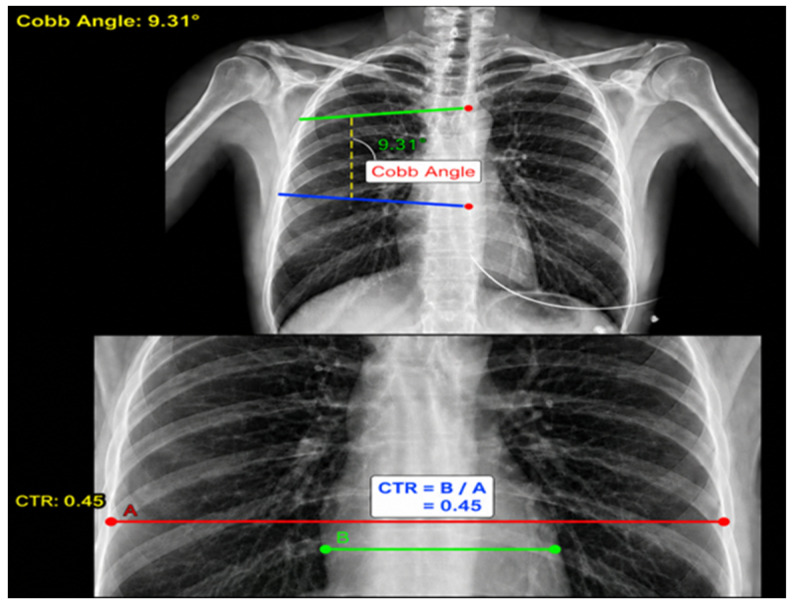
Calculation of the Cardiothoracic Ratio (CTR) and Cobb Angle.

In the present study, the Cobb angle was not intended for the clinical diagnosis or grading of scoliosis. Instead, it was used as an image-derived quantitative descriptor representing thoracic spinal alignment visible on the available portable frontal chest radiographs. Measurements were performed only on vertebral segments that were clearly identifiable within the radiographic field of view, and the resulting values were incorporated exclusively as predictive imaging features in the machine learning models. A representative example of the measurement procedure is provided in Figure 1.

Because portable chest radiographs obtained in the intensive care unit are acquired primarily for thoracic assessment rather than scoliosis evaluation, the automatically calculated Cobb angle should not be interpreted as a conventional diagnostic scoliosis measurement. Instead, it was considered a standardized quantitative descriptor of thoracic spinal alignment that could be extracted consistently from all radiographs using the same automated algorithm and preprocessing pipeline. Accordingly, the Cobb angle was included as an imaging-derived quantitative feature for comparative machine learning analysis rather than as a diagnostic measure of spinal deformity.

#### 2.3.2. Cardiothoracic Ratio Calculation

The cardiothoracic ratio (CTR) is defined as the ratio of the maximum transverse cardiac diameter to the maximum internal transverse diameter of the thoracic cavity measured on a posteroanterior (PA) chest radiograph [6,15,18]. CTR is one of the most widely used radiographic indices for assessing cardiac enlargement and has long been employed as a simple and practical indicator of cardiomegaly in clinical practice [6,12].

In the present study, CTR was calculated by dividing the maximum cardiac width by the maximum internal thoracic width measured on the radiograph portable frontal chest radiograph. A CTR value greater than 0.50 was considered indicative of cardiomegaly [18,22]. Figure 1 illustrates the measurements used for calculating both the cardiothoracic ratio and the Cobb angle. Because portable frontal chest radiographs may be affected by projection-related cardiac magnification, the calculated CTR values were interpreted as standardized image-derived variables for machine learning analysis rather than as definitive diagnostic measurements of cardiomegaly.

The formula used for calculating the cardiothoracic ratio (CTR) is presented below:

$$CTR=\frac{Cardiac\;Width}{Thoracic\;Width}$$

### 2.4. Software and Statistical Analysis

All image preprocessing and feature extraction procedures were performed using Python 3.12, employing the Scikit-image (skimage), SciPy, and Mahotas libraries. All machine learning analyses, feature selection procedures, and model evaluations were conducted using the MATLAB Online platform (MathWorks, Natick, MA, USA).

To investigate the effects of feature dimensionality and feature selection strategies on ICU mortality prediction, two complementary analyses were performed. Experiment Set-1 utilized a clinically interpretable feature set consisting of 12 radiographic and demographic variables. Experiment Set-2 employed a high-dimensional feature space composed of 74 radiographic, radiological, and image-derived features.

For the clinically interpretable feature set, feature relevance was evaluated using the Minimum Redundancy Maximum Relevance (MRMR) algorithm and Permutation Importance analysis. Feature subsets of varying dimensionality were subsequently generated and compared to determine whether predictive performance could be maintained using a reduced number of variables.

For the high-dimensional feature space, six feature selection methods were comparatively evaluated: Analysis of Variance (ANOVA), Chi-Square, Kruskal–Wallis, Minimum Redundancy Maximum Relevance (MRMR), ReliefF, and Shapley-based importance analysis. The top-ranked features identified by each method were compared to evaluate feature selection consistency. Features that were consistently identified across multiple feature selection methods within the 74-feature experimental framework were designated as the consensus feature subset for Experiment Set-2. This terminology distinguishes these features from the reduced four-feature subsets generated independently within Experiment Set-1. This approach enabled the investigation of feature stability and the identification of consistently selected predictors associated with mortality. For transparency, the comparative ranking of the top 15 features identified by each feature selection method is provided in Supplementary Table S3.

For all classification analyses, mortality was consistently defined as the positive class (Class 0), whereas survival was defined as the negative class (Class 1). Consequently, sensitivity (true positive rate) was calculated based on the correct identification of mortality cases, while specificity (true negative rate) was calculated based on the correct identification of survivors. All confusion matrix interpretations and performance metrics were reviewed to ensure consistency with this class definition.

Model performance was evaluated using the area under the receiver operating characteristic curve (AUC), sensitivity, specificity, accuracy, and F1-score [10,13]. To prevent information leakage and ensure unbiased evaluation, all feature selection procedures were performed. All performance metrics were reported using consistent definitions throughout the manuscript. Area under the receiver operating characteristic curve (AUC) is presented as a value between 0 and 1, whereas sensitivity, specificity, accuracy, precision, recall, and F1-score are reported as percentages (%). All performance metrics were reported using consistent definitions throughout the manuscript. Area under the receiver operating characteristic curve (AUC) is presented as a value between 0 and 1, whereas sensitivity, specificity, accuracy, precision, recall, and F1-score are reported as percentages (%). All percentage values were rounded to one decimal place for consistency across tables, figures, and text. To prevent information leakage and ensure unbiased evaluation, all feature selection procedures were performed exclusively within the training dataset.

Furthermore, five-fold cross-validation (k = 5) was implemented within the training set to optimize model parameters and assess model robustness. The independent test set was excluded from feature selection, model training, algorithm comparison, and parameter optimization procedures and was reserved solely for final performance evaluation. To eliminate any possibility of data leakage, all feature selection procedures, including MRMR, Permutation Importance, ANOVA, Chi-Square, Kruskal–Wallis, ReliefF, and Shapley-based importance analysis, were performed exclusively using the training dataset. The independent test dataset remained completely isolated throughout feature selection, feature ranking, model development, algorithm comparison, and hyperparameter optimization, and was accessed only once for the final performance evaluation of the selected models.

Among the validation strategies considered in this study, the independent test set and five-fold cross-validation were regarded as the primary approaches for performance evaluation. Resubstitution analysis was included only to provide an exploratory comparison of model behavior under different feature selection strategies. Both experimental frameworks were evaluated using the same independent hold-out test dataset (400 training/100 testing), thereby ensuring a consistent basis for performance comparison. In addition, five-fold cross-validation and resubstitution analyses were performed as complementary validation procedures where appropriate to assess model robustness and methodological consistency. Regardless of the validation strategy, all comparisons between Experiment Set-1 and Experiment Set-2 reported in this study are based on results obtained from the same independent test dataset.

To facilitate reproducibility, all analytical procedures, including feature extraction, feature selection, model development, validation, and performance evaluation, followed the workflow described in the Methods section. The analysis scripts supporting the findings of this study are available from the corresponding author upon reasonable request.

### 2.5. Overall Experimental Workflow

**Figure 2:**
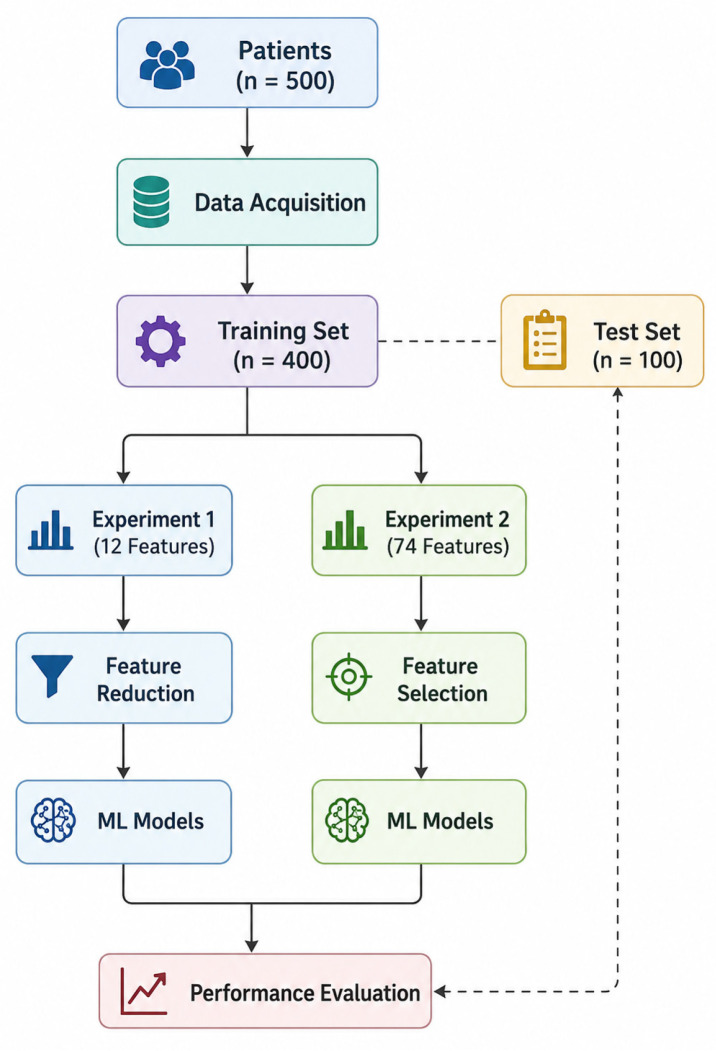
Schematic overview of the experimental workflow for model development and evaluation.

The overall study workflow is summarized in Figure 2. After dataset preparation, the 500 patients were divided into 400 training cases and 100 independent test cases. The independent test dataset remained completely isolated throughout feature selection, model development, algorithm comparison, and parameter optimization.

Schematic overview of the proposed experimental framework. The dataset was divided into independent training and test sets. Two complementary experimental frameworks were performed using the same independent test dataset. Experiment Set-1 investigated clinically interpretable features together with feature reduction techniques, whereas Experiment Set-2 evaluated multiple feature selection methods in a high-dimensional feature space. Finally, all models were compared using identical performance metrics. The study consisted of two complementary experimental frameworks. Experiment Set-1 investigated whether clinically interpretable radiographic and demographic variables could provide reliable mortality prediction. This framework evaluated the complete 12-feature dataset together with multiple reduced feature subsets generated using MRMR and Permutation Importance analyses.

Experiment Set-2 focused on a high-dimensional feature space comprising 74 radiographic, radiological, and image-derived features. Six feature selection methods (ANOVA, Chi-Square, Kruskal–Wallis, MRMR, ReliefF, and Shapley-based importance analysis) were comparatively evaluated to investigate feature stability, feature selection consistency, and predictive performance.

Although different feature selection strategies were investigated within the two experimental frameworks, all final model performances were evaluated using the same independent test dataset, thereby ensuring a fair and unbiased comparison between experiments.

## 3. Analysis of Feature Selection Strategies and Analyses

A total of 500 chest radiographs obtained from patients admitted to the Internal Medicine Intensive Care Unit were included in the study. All radiographs were acquired within the first 24 h of ICU admission, and mortality prediction models were subsequently developed using machine learning techniques. The study population consisted of non-COVID adult patients (>18 years) hospitalized between 2022 and 2023.

Following the workflow presented in Figure 2. The first analysis was based on a clinically interpretable feature set consisting of 12 radiographic and demographic variables, including cardiomegaly, unilateral and bilateral infiltrates, unilateral and bilateral pleural effusions, pneumothorax, calcified aorta, cardiothoracic ratio (CTR), Cobb angle, age, blood group, and gender. The objective of this analysis was to investigate whether reliable mortality prediction could be achieved using a limited number of clinically meaningful features.

The second analysis was conducted using a high-dimensional feature space consisting of 74 radiographic, radiological, and image-derived features. This analysis was designed to evaluate the effects of different feature selection strategies on predictive performance and feature stability. Six feature selection methods, namely ANOVA, Chi-Square, Kruskal–Wallis, MRMR, ReliefF, and Shapley-based importance analysis, were comparatively assessed. The selected feature subsets were subsequently used for machine learning model development and performance evaluation. The comparative ranking of the top 15 features identified by each feature selection method is presented in Supplementary Table S3.

Machine learning models were trained and evaluated using multiple validation approaches, including hold-out validation, five-fold cross-validation, and resubstitution analysis. The primary objective was not only to identify high-performing prediction models but also to investigate the relationship between feature dimensionality, feature selection strategies, and model interpretability. Unless otherwise stated, the interpretation of model performance throughout the Results section is based primarily on the independent test set and cross-validation results, whereas the resubstitution analysis is presented solely as supplementary exploratory information. For consistency, all direct comparisons between the two experimental frameworks presented in this study refer to the performance obtained on the same independent hold-out test dataset.

The baseline demographic and clinical characteristics of the study population are summarized in Table 1. Age distribution, sex, blood group categories, and available clinical variables were evaluated to characterize the study cohort prior to machine learning analyses.

### 3.1. Analyses Performed with 12 Features

Bagged Trees: The results of this model indicate that although it demonstrated relatively limited performance during the validation phase, its performance improved significantly on the test set. While the validation accuracy was 0.76, the accuracy increased to 88.0% on the independent test set, with the error rate remaining at 0.12. This suggests that the model achieved strong generalization performance on the test data. This outcome indicates that the ensemble approach can produce more stable results by combining the strengths of different learners.

A more detailed evaluation of the class-based results showed that the precision for the mortality class (Class 0) reached 1, whereas its sensitivity was 0.76, indicating that although every patient predicted as mortality was correctly classified, 12 mortality cases were misclassified as survival. In contrast, the survival class (Class 1) achieved a specificity of 1, indicating that none of the surviving patients was incorrectly classified as mortality. Furthermore, the F1-score of 0.86 for the mortality class demonstrates a strong balance between precision and recall.

**Figure 3:**
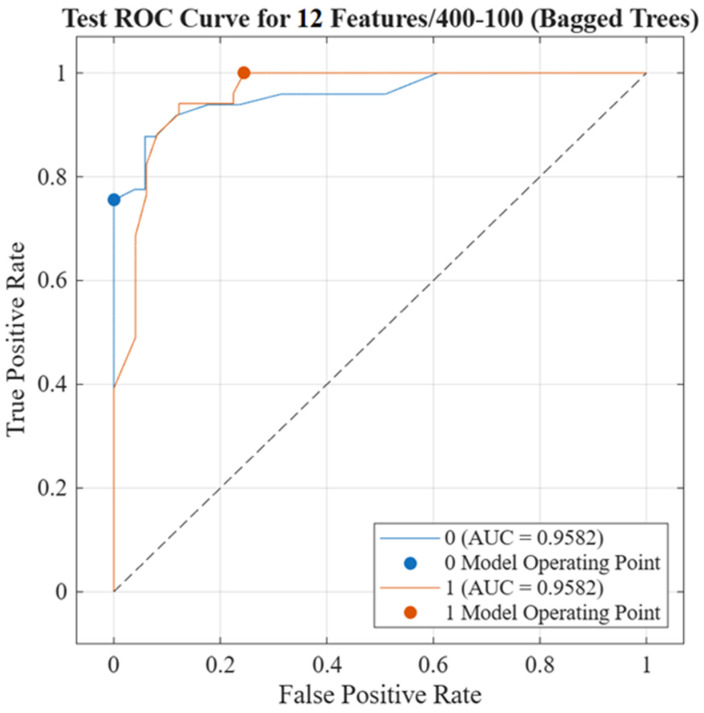
The ROC curve for 12 features (Bagged trees). Receiver Operating Characteristic (ROC) curves were generated using MATLAB Classification Learner. In accordance with the standard ROC representation, the vertical axis represents the True Positive Rate (Sensitivity), whereas the horizontal axis represents the False Positive Rate (1 − Specificity). Therefore, lower values on the horizontal axis indicate higher model specificity.

**Figure 4:**
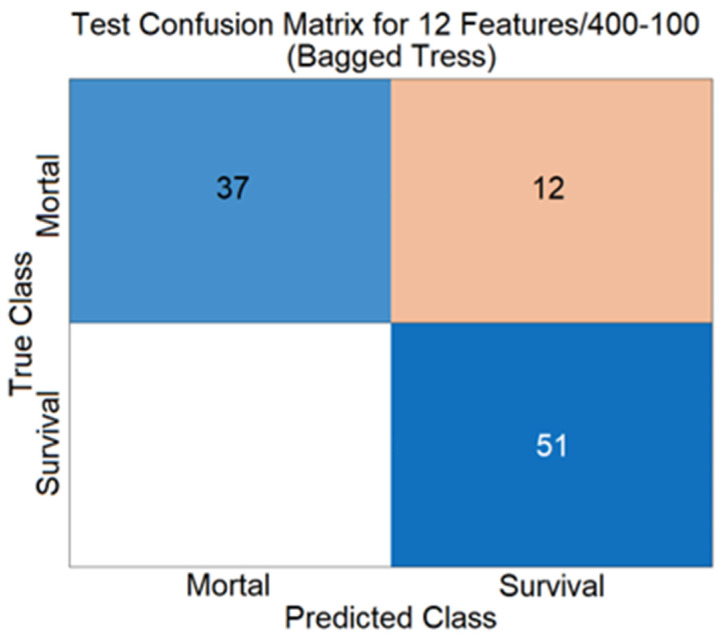
The Confusion Matrix for 12 features (Bagged trees).

Figure 3 and Figure 4 present the performance evaluation of the Bagged Trees ensemble classifier using the complete set of 12 features. Figure 3 illustrates the receiver operating characteristic (ROC) curve obtained from the test data, highlighting the trade-off between sensitivity and specificity; the area under the curve (AUC) of 0.96 indicates discriminative performance. Figure 4 shows the corresponding confusion matrix for the test dataset, summarizing the distribution of correct and incorrect predictions across all classes and demonstrating the model’s performance in distinguishing survival categories. The comparative performances of the next two best-performing algorithms (Boosted Trees and RUSBoosted) are summarized in Table 2.

Among the evaluated ensemble learning algorithms using all 12 features, the Bagged Trees classifier achieved the best overall performance (Table 2). It yielded an AUC of 0.95, a sensitivity of 0.76, a specificity of 1.00, an accuracy of 0.88, and an F1-score of 0.86, demonstrating the strongest discriminative ability and overall classification performance among the evaluated models.

The Boosted Trees classifier also produced competitive results, achieving an AUC of 0.92, a sensitivity of 0.65, a specificity of 0.98, an accuracy of 0.82, and an F1-score of 0.81. Although its specificity remained high, the lower sensitivity indicated a reduced ability to identify mortality cases compared with Bagged Trees.

**Figure 5:**
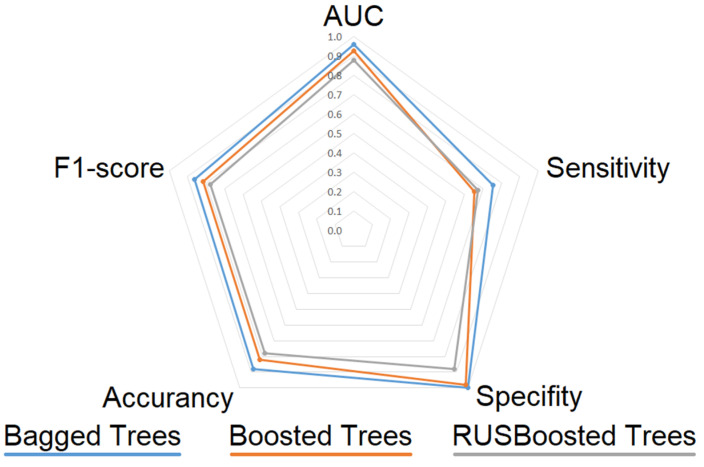
Comparison Table (Algorithms Highlighted in the Analysis with 12 features).

The RUSBoosted classifier achieved an AUC of 0.87, a sensitivity of 0.67, a specificity of 0.88, an accuracy of 0.78, and an F1-score of 0.78. While its overall performance was lower than that of the other ensemble models, it still provided acceptable classification performance. Figure 5 presents a radar-chart comparison of the key performance metrics for all three algorithms.

To identify the most informative predictors, feature importance was evaluated using two complementary feature selection approaches: Minimum Redundancy Maximum Relevance (MRMR) and Permutation Importance. These methods were employed to assess the contribution of individual features to mortality prediction while reducing redundancy among predictors.

**Figure 6:**
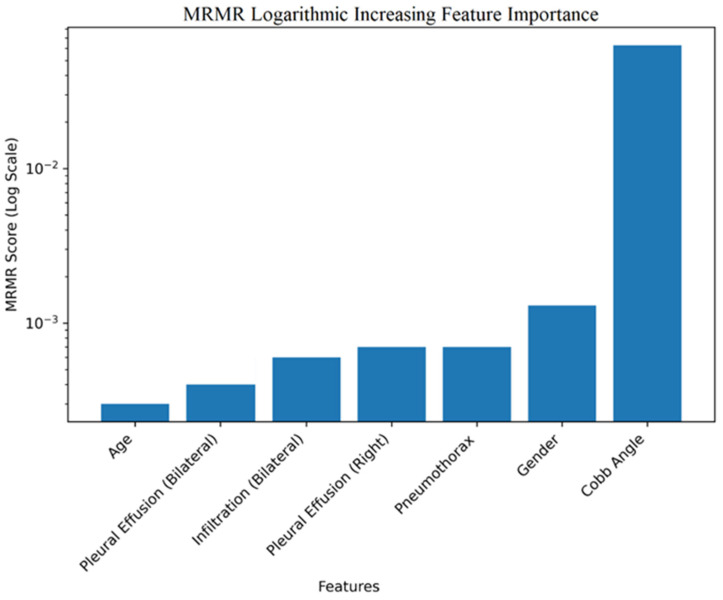
MRMR feature importance graphic.

**Figure 7:**
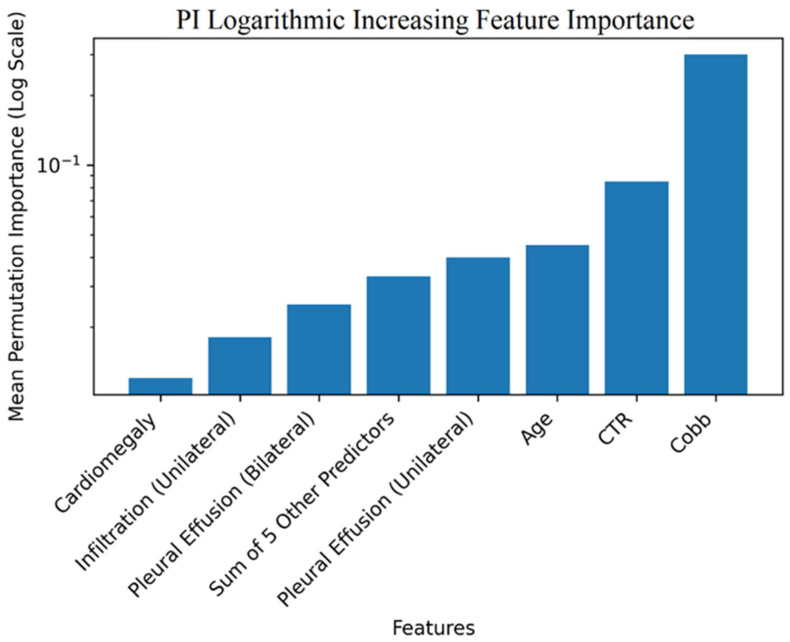
Permutation importance (PI) and feature importance graphic.

Figure 6 presents the feature importance rankings obtained using the MRMR algorithm. The results indicate that a limited subset of features contributed disproportionately to model performance, supporting the feasibility of feature reduction without substantial loss of predictive accuracy. The corresponding feature importance results obtained from the Permutation Importance analysis are presented in Figure 7.

At this stage, analyses were conducted separately for the top four features identified using the MRMR and Permutation Importance feature selection methods. Based on these four most significant features, additional analyses were performed, and the outputs of the three best-performing algorithms obtained from these analyses are presented below.

### 3.2. Analyses Performed with 4 Features Obtained Using the MRMR Algorithm

**Figure 8:**
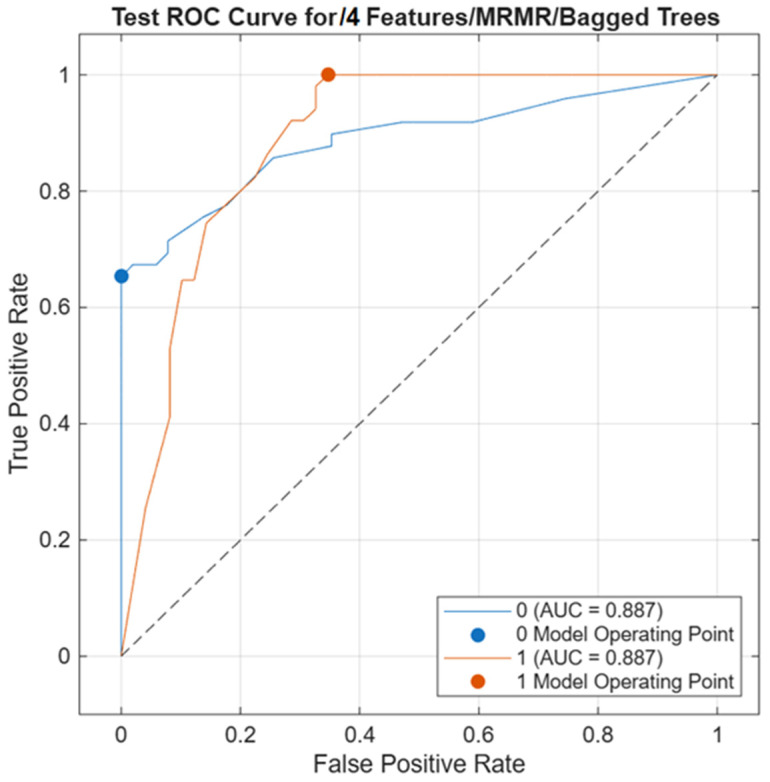
The ROC curve for 4 features (Bagged trees).

**Figure 9:**
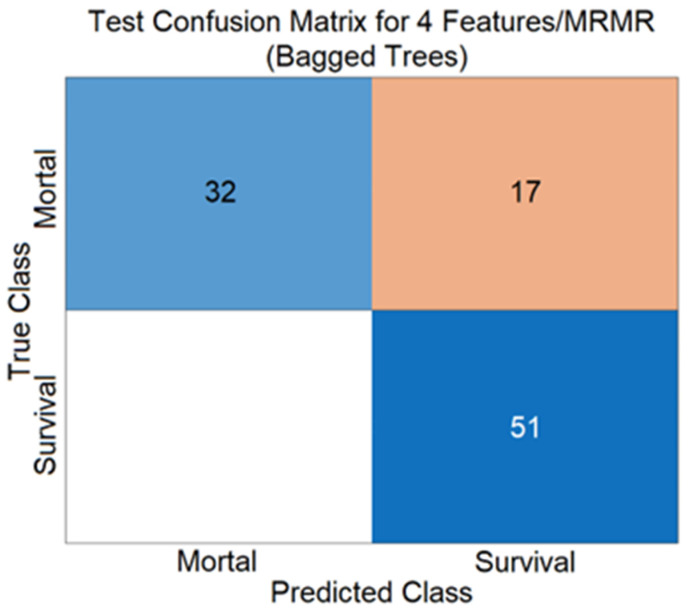
The Confusion matrix for 4 features (Bagged trees).

Bagged Trees: The Bagged Trees model achieved an AUC of 0.88. Figure 8 and Figure 9 present the performance of the Bagged Trees classifier using the top four features selected via the MRMR method, showing the ROC curve and the corresponding confusion matrix, respectively.

Using the four-feature subset selected by the MRMR algorithm, the Bagged Trees classifier again achieved the best overall performance (Table 3). It yielded an AUC of 0.88, a sensitivity of 0.65, a specificity of 1.00, an accuracy of 0.83, and an F1-score of 0.79, indicating that competitive classification performance was maintained despite the substantial reduction in the number of input features.

The Fine Tree classifier achieved an AUC of 0.82, a sensitivity of 0.78, a specificity of 0.96, an accuracy of 0.78, and an F1-score of 0.77. Compared with Bagged Trees, it showed improved sensitivity but lower specificity and overall discriminative performance.

**Figure 10:**
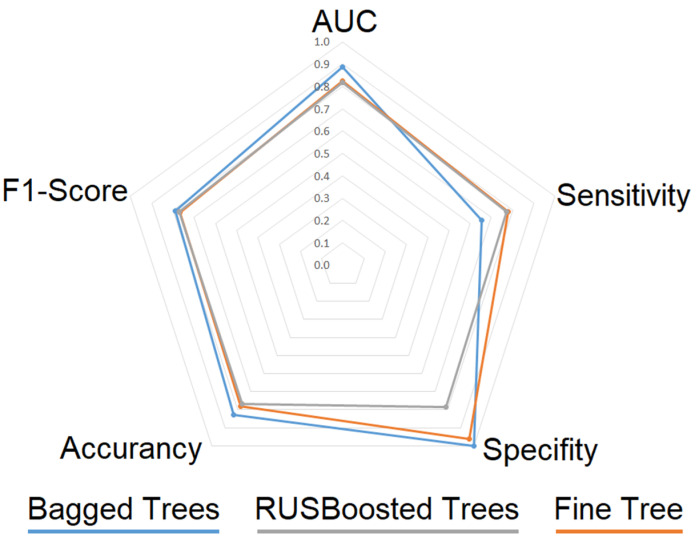
Radar-chart comparison of the three best-performing algorithms using the four-feature subset selected by the MRMR method.

The RUSBoosted Trees classifier achieved an AUC of 0.81, a sensitivity of 0.77, a specificity of 0.78, an accuracy of 0.77, and an F1-score of 0.77. Although its overall performance was slightly lower than that of the other two classifiers, it produced consistent classification results using only four selected features. Figure 10 presents a radar-chart comparison of the key performance metrics for all three algorithms.

The analysis performed using the four most informative features selected by the MRMR algorithm demonstrated that substantial feature reduction could be achieved while maintaining satisfactory predictive performance. Among the evaluated machine learning models, the Bagged Trees classifier achieved the highest performance, yielding an AUC of 0.88. These findings suggest that the selected feature subset contains robust prognostic information and that reliable ICU mortality prediction can be achieved using a limited number of clinically relevant variables.

### 3.3. Analyses Performed with 4 Features Obtained Using the MRMR and Permutation Importance Methods

In this analysis, the four common features identified by both the MRMR and Permutation Importance methods were used to develop the classification models. Among the evaluated algorithms, the three best-performing classifiers were selected for further analysis based on their predictive performance.

**Figure 11:**
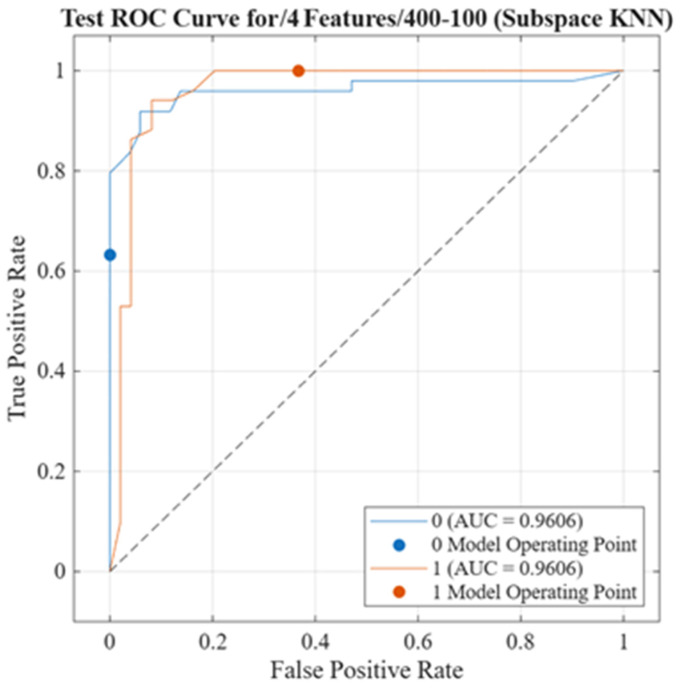
The ROC curve for 4 features (Subspace KNN).

**Figure 12:**
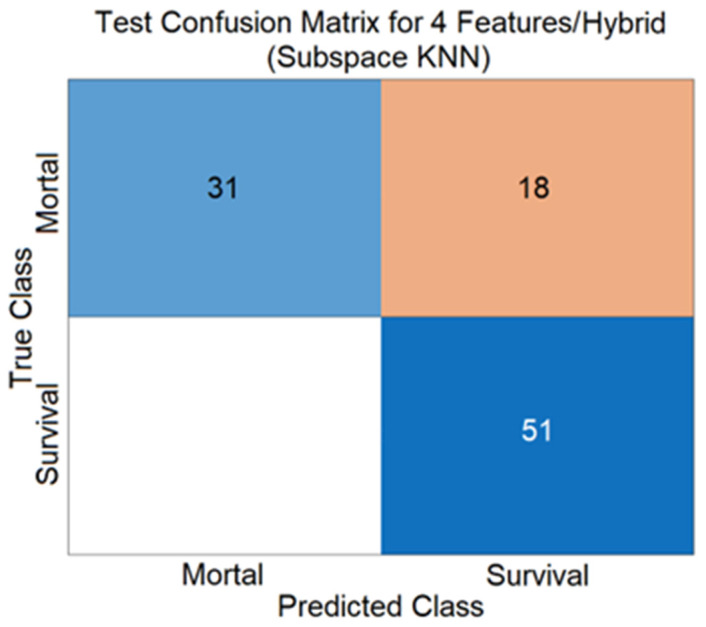
The Confusion matrix for 4 features (Subspace KNN).

The Subspace KNN classifier achieved the best overall performance using the four selected features. During the validation phase, the model achieved an accuracy of 0.70, while its accuracy increased to 0.82 on the independent test set, corresponding to a test error rate of 0.18. The model was constructed using 30 sub-learners operating in three-dimensional feature subspaces. Despite relying on only four input features, the classifier maintained high predictive performance without requiring additional dimensionality reduction techniques, indicating that the selected features provided substantial discriminative information for the classification task. The default cost matrix was used throughout the analysis. Figure 11 and Figure 12 present the ROC curve and the corresponding confusion matrix of the Subspace KNN classifier obtained using the four selected features.

Boosted Trees and Bagged Trees were also evaluated using the four features selected by the hybrid feature selection approach. As summarized in Table 4, the Boosted Trees classifier achieved an AUC of 0.93, a sensitivity of 0.55, a specificity of 0.98, an accuracy of 0.77, and an F1-score of 0.76. Although the classifier maintained high specificity and good discriminative capability, its lower sensitivity reduced its overall performance compared with the Subspace KNN classifier.

**Figure 13:**
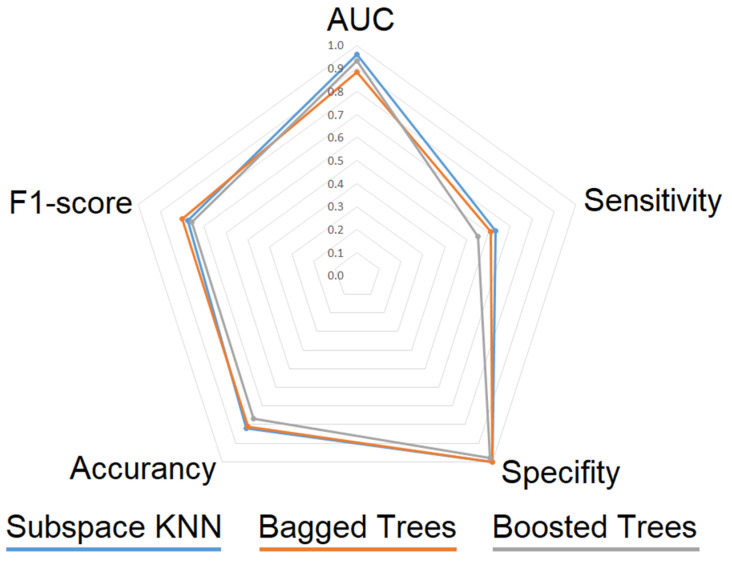
Comparison Table (Algorithms Highlighted in the Analysis with 4 Features with hybrid system).

Similarly, the Bagged Trees classifier achieved an AUC of 0.88, a sensitivity of 0.61, a specificity of 1.00, an accuracy of 0.81, and an F1-score of 0.80. The model demonstrated strong overall classification performance and perfect specificity, although its sensitivity remained lower than that of an ideal mortality prediction model. Overall, Subspace KNN achieved the best predictive performance among the evaluated classifiers using the four selected features. Figure 13 presents a radar-chart comparison of the key performance metrics for the three algorithms.

When the MRMR and Permutation Importance methods are evaluated together, it is observed that the variables Cobb angle, age, bilateral pleural effusion, and unilateral infiltration consistently emerge as the most prominent features in both methods. This finding indicates that these features act as consistent and strong predictors of mortality independent of the specific model used. Using these four core features, additional analyses were conducted, and the performance of the algorithms was further evaluated.

### 3.4. Analyses Performed with 4 Features Obtained Using the Permutation Importance Methods

Weighted KNN achieved the highest discriminative capability among the evaluated classifiers when the four features selected by the Permutation Importance method were used. The classifier yielded an AUC of 0.98, indicating excellent discrimination between mortality and survival despite the substantial reduction in feature dimensionality.

**Figure 14:**
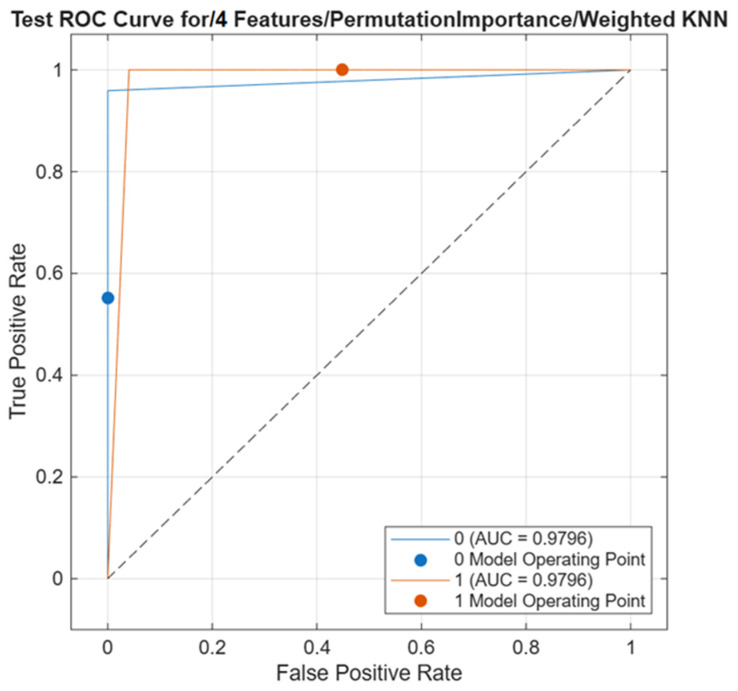
The ROC curve for 4 features (Weighted KNN).

**Figure 15:**
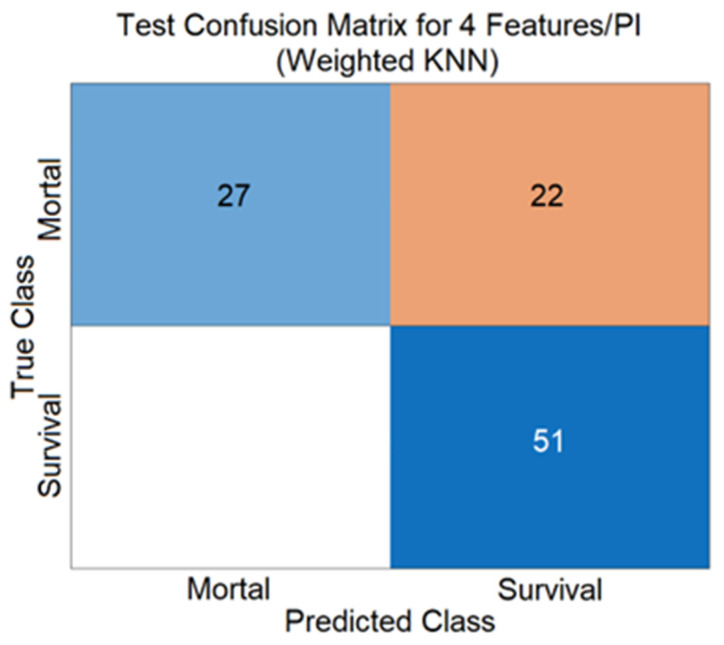
The Confusion matrix for 4 features (Weighted KNN).

The model was evaluated on the independent test dataset, achieving an accuracy of 0.78 with a corresponding test error rate of 0.22. These findings demonstrate that a limited set of informative features can preserve strong discriminative performance while maintaining a simple and computationally efficient classification model. Figure 14 and Figure 15 present the ROC curve and the corresponding confusion matrix of the Weighted KNN classifier obtained from the independent test dataset.

Bagged Trees and RUSBoosted Trees were also evaluated using the four features selected by the Permutation Importance method. As summarized in Table 5, the Bagged Trees classifier achieved an AUC of 0.93, a sensitivity of 0.82, a specificity of 0.82, an accuracy of 0.82, and an F1-score of 0.82, demonstrating strong and well-balanced classification performance.

Similarly, the RUSBoosted Trees classifier achieved an AUC of 0.90, while maintaining a sensitivity of 0.82, a specificity of 0.82, an accuracy of 0.82, and an F1-score of 0.82. Although its overall classification performance was comparable to that of Bagged Trees, its lower AUC indicated reduced discriminative capability.

**Figure 16:**
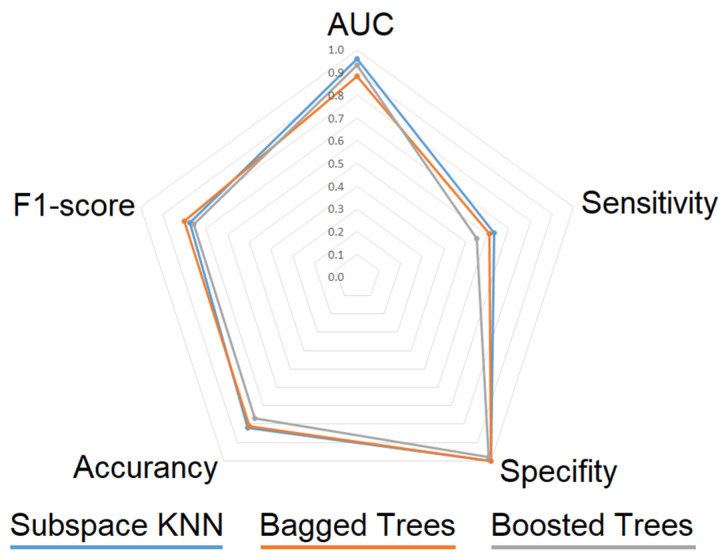
Comparison Table (Algorithms Highlighted in the Analysis with 4 Features with permutation importance).

Table 5 summarizes the comparative performance of the three best-performing classifiers using the four features selected by the Permutation Importance method. Among the evaluated models, Weighted KNN achieved the highest AUC (0.98), indicating the strongest discriminative capability, whereas Bagged Trees and RUSBoosted Trees provided more balanced classification performance with identical sensitivity, specificity, accuracy, and F1-score values. The comparative performance of the evaluated machine learning algorithms is shown in Figure 16.

### 3.5. Analyses Performed with 74 Features

#### 3.5.1. Selection and Validation Strategy 1 (Cross Validation)

To investigate the effects of feature selection strategies on ICU mortality prediction, a high-dimensional dataset consisting of 74 radiographic, radiological, and image-derived features was analyzed. The dataset included 400 training cases and 100 independent test cases. The extracted features comprised radiological findings evaluated by an experienced radiologist, as well as quantitative image-based descriptors derived from chest radiographs.

A multi-stage feature selection framework was adopted to identify the most informative predictors while reducing feature redundancy and model complexity. In addition, multiple validation strategies were employed to evaluate model robustness and generalizability.

Three validation approaches were investigated: hold-out validation, k-fold cross-validation (k = 2, 5, and 20), and resubstitution analysis. Each validation strategy provides complementary information regarding model behavior. Hold-out validation enables unbiased evaluation using an independent test set, cross-validation provides estimates of model stability across different training subsets, and resubstitution analysis offers an exploratory assessment of the learning capacity of the evaluated algorithms.

The inclusion of multiple k-fold configurations (k = 2, 5, and 20) allowed the robustness of model performance to be assessed under different cross-validation settings.

The combined use of these validation approaches enabled a comprehensive assessment of predictive performance, model stability, and feature selection consistency. This framework was adopted to ensure that the identified feature subsets and resulting machine learning models were not dependent on a single validation strategy.

Coarse KNN was evaluated using the complete set of 74 features. The classifier achieved an accuracy of 0.76 on both the validation and independent test datasets, indicating stable generalization performance.

**Figure 17:**
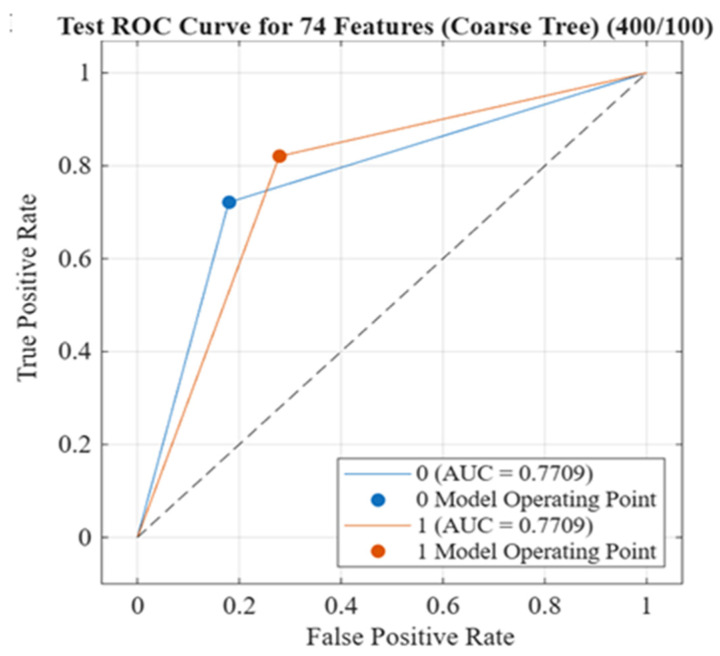
The ROC curve for 74 features (Coarse Tree).

Class-specific analysis revealed higher precision for the survival class and higher recall for the mortality class, suggesting that the model was reasonably effective in identifying mortality cases while maintaining acceptable overall discrimination. Although the model demonstrated stable performance, it was subsequently outperformed by other algorithms and feature selection strategies evaluated in this study. Figure 17 presents the ROC curve of the Coarse Tree classifier based on the 74-feature dataset.

**Figure 18:**
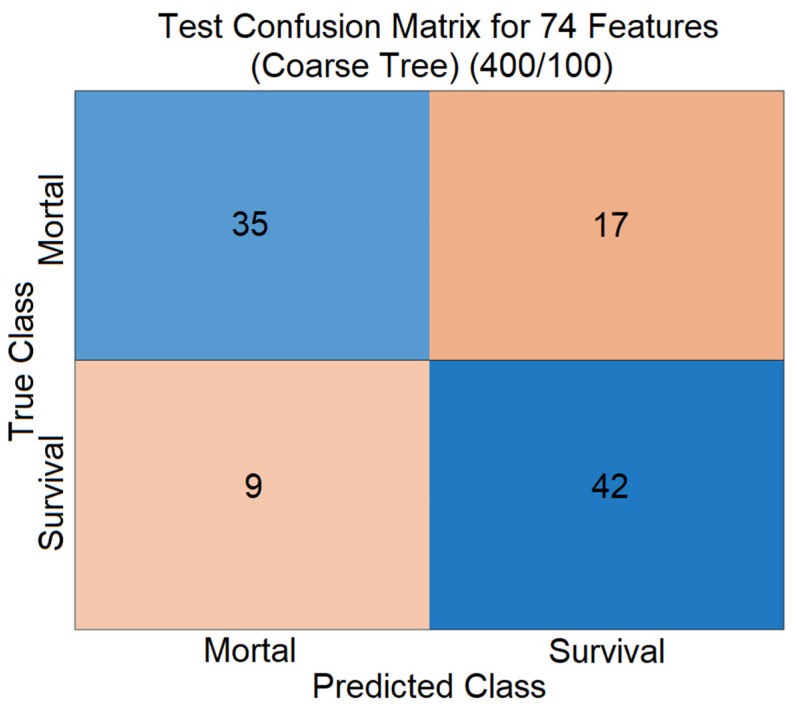
The Confusion matrix for 74 features (Coarse Tree).

**Figure 19:**
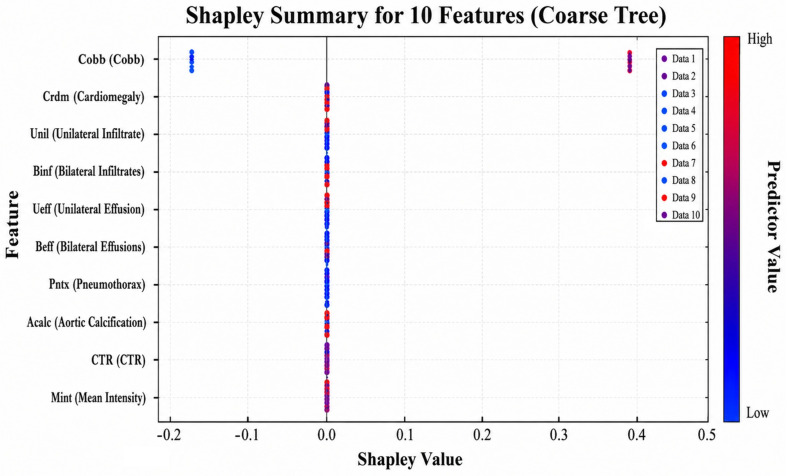
Shapley Summary for 10 Features (Coarse Tree).

**Figure 20:**
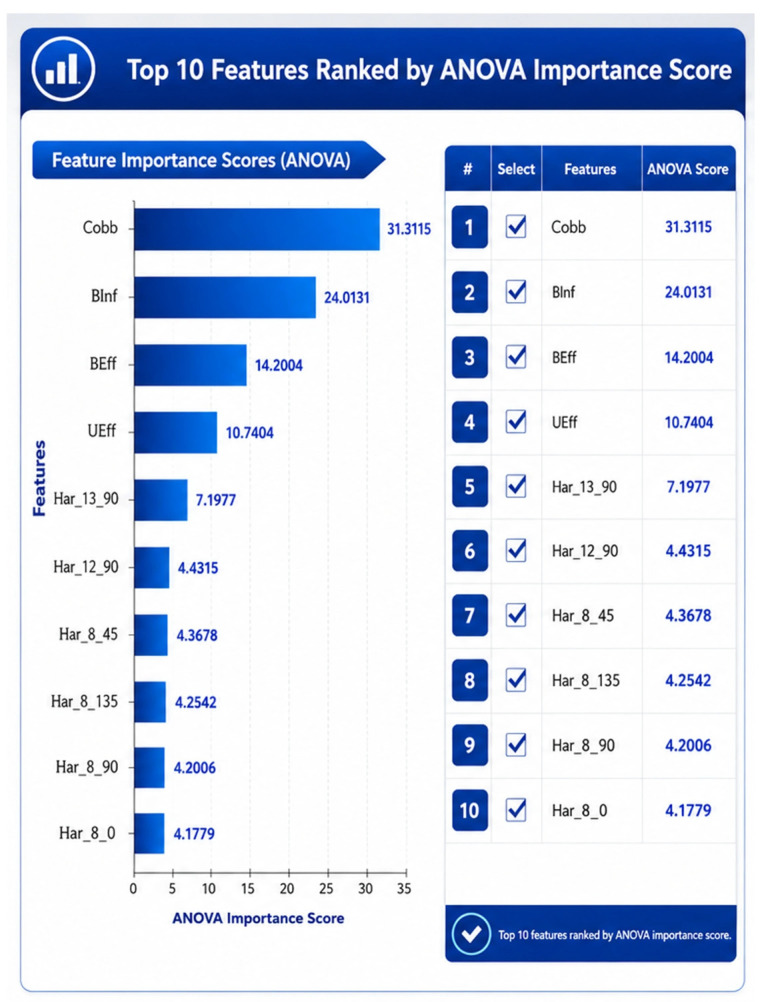
Anova algorithm for 10 Features (Coarse Tree).

To investigate the relative importance of individual predictors, feature ranking analyses were performed using Shapley-based importance estimation and the ANOVA feature selection method. The confusion matrix of the Coarse Tree classifier using the 74-feature dataset is presented in Figure 18. Figure 19 presents the Shapley summary plot obtained from the complete 74-feature dataset, highlighting the contribution of the most influential predictors to model performance. Figure 20 presents the corresponding ANOVA-based feature ranking results.

The rankings obtained from different feature selection approaches demonstrated that only a limited subset of predictors consistently contributed to mortality prediction. This observation supports the hypothesis that substantial feature reduction may be achieved without a major loss in predictive performance.

**Figure 21:**
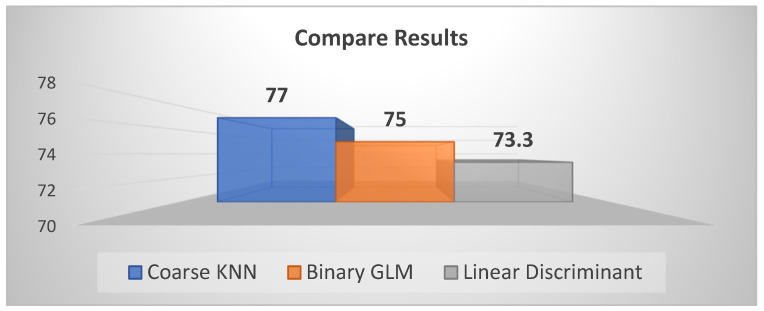
Compare Results (Coarse Tree).

Figure 21 presents the comparative performance of the best-performing machine learning algorithms evaluated using the complete feature set. Among the evaluated models, Coarse KNN achieved the highest overall performance and was therefore selected as the reference model for subsequent feature selection analyses. Figure 19 presents the SHAP summary plot for the 10-feature subset obtained using the Coarse Tree classifier.

Figure 21 presents the comparative performance of the three best-performing machine learning algorithms evaluated using the complete 74-feature dataset.

Binary GLM logistic regression and Linear Discriminant Analysis were also evaluated using the complete 74-feature dataset.

A comparative summary of the best-performing algorithms evaluated using the complete 74-feature dataset is presented in Table 6. The results indicate that Coarse KNN achieved the highest overall performance and was therefore selected as the reference model for subsequent feature selection analyses.

#### 3.5.2. Selection and Validation Strategy 2 (Hold-Out Validation)

In addition to cross-validation, hold-out validation was employed to further assess model performance and generalizability. In this approach, the dataset was partitioned into separate training and validation subsets, allowing model performance to be evaluated on previously unseen data.

The hold-out strategy was used to investigate the sensitivity of machine learning algorithms to a fixed data partition, to assess the potential impact of distributional differences between training and validation subsets, and to provide an evaluation framework that more closely resembles real-world clinical deployment scenarios.

The results obtained from hold-out validation demonstrated that certain algorithms exhibited performance reductions compared with cross-validation analyses. This observation is likely related to the limited dataset size and class distribution characteristics. Nevertheless, the hold-out approach provided valuable complementary information regarding model robustness and generalizability, supporting a more comprehensive interpretation of algorithm performance. The performance metrics obtained from the hold-out validation analyses are summarized in Table 7.

#### 3.5.3. Selection and Validation Strategy 3 (Resubstitution Validation)

In addition to hold-out and cross-validation approaches, resubstitution analysis was performed as an exploratory tool to investigate algorithm behavior and feature selection consistency. Because resubstitution evaluates models using the same data employed for training, the resulting performance estimates were not considered as final measures of model effectiveness. Instead, this approach was used to compare feature selection strategies and to identify stable predictors of ICU mortality.

Among the evaluated algorithms, Bagged Trees demonstrated the highest performance under the resubstitution framework and was therefore selected as the reference model for subsequent feature selection analyses. As expected, the model achieved near-perfect performance on the training data, reflecting the optimistic nature of resubstitution estimates. Consequently, these results were interpreted only in the context of feature ranking and algorithm comparison rather than model generalizability.

Six feature selection methods were subsequently evaluated: ANOVA, Chi-Square, Kruskal–Wallis, MRMR, ReliefF, and Shapley-based importance analysis. For each method, the 15 highest-ranked features were identified and compared.

**Figure 22:**
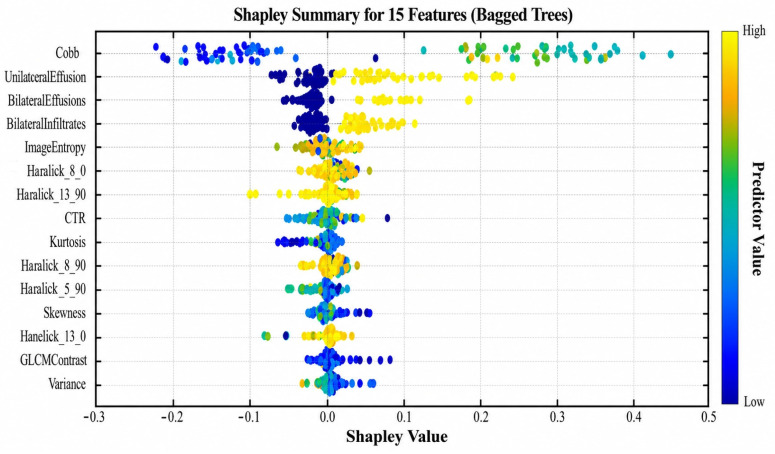
Shapley Summary for 15 Features (Coarse Tree-Resubstituion Validation).

Figure 22 presents the Shapley Additive Explanations (SHAP) analysis summary plot obtained from the Bagged Trees model, illustrating the relative contribution of the most influential predictors to mortality prediction. Higher absolute SHAP values indicate a greater contribution of the corresponding feature to the model output.

**Figure 23:**
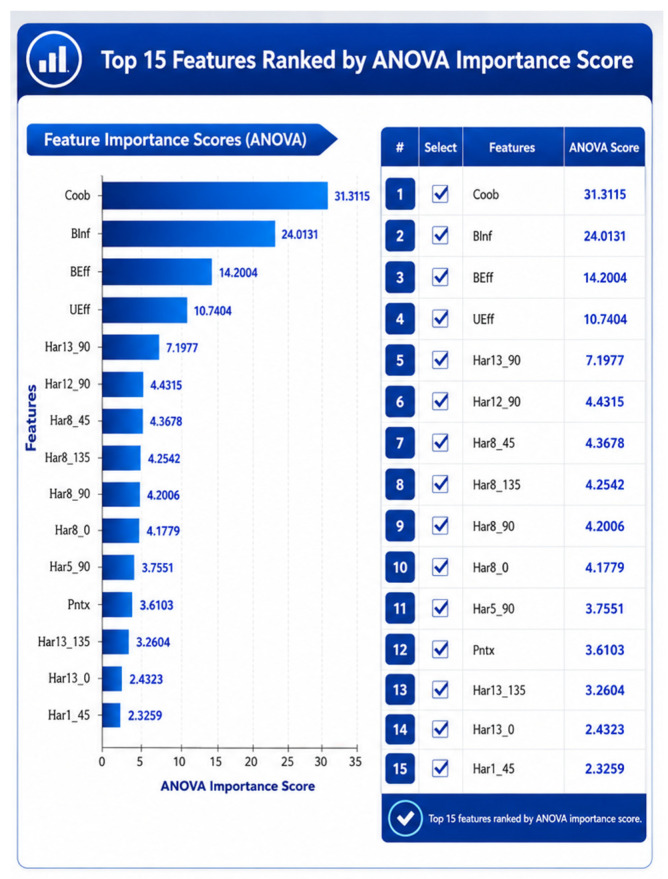
Anova algorithm for 15 Features (Coarse Tree-Resubstituion Validation).

Figure 23 presents the feature ranking generated by the ANOVA feature selection method, illustrating the relative discriminative ability of individual predictors within the complete 74-feature space.

Despite methodological differences, several predictors consistently appeared among the top-ranked variables across multiple approaches. The comparison of feature rankings revealed four common predictors that were selected by all feature selection methods: Cobb angle, bilateral infiltrates, bilateral pleural effusion, and unilateral pleural effusion. These variables were defined as the consensus feature subset because of their consistent association with mortality prediction.

The rankings obtained from the different feature selection methods are summarized in Table 8. Among the evaluated feature selection strategies, MRMR and Kruskal–Wallis produced the highest-performing feature subsets, whereas ANOVA, Chi-Square, ReliefF, and Shapley-based selection yielded comparable but slightly lower performance levels.

The identification of a common four-feature subset across all methods suggests that robust mortality prediction may be achieved using a substantially reduced and clinically interpretable feature set.

#### 3.5.4. Overall Evaluation of Validation and Feature Selection Strategies

The combination of cross-validation, hold-out validation, and resubstitution analyses enabled a comprehensive assessment of model performance, robustness, and feature selection stability. Each validation strategy provided complementary information regarding algorithm behavior and contributed to a more reliable interpretation of the obtained results. This consistency suggests that these predictors are robust with respect to the feature selection algorithm employed.

Among the evaluated feature selection approaches, MRMR and Kruskal–Wallis produced the highest-performing feature subsets, while the common four-feature core set identified across all methods achieved comparable predictive performance despite substantial dimensionality reduction. The results suggest that a limited number of clinically interpretable radiographic features may retain most of the predictive information required for ICU mortality prediction.

It should be emphasized that resubstitution analysis was not used to estimate final model performance. Because resubstitution evaluates a model on the same data used for training, the resulting performance estimates are inherently optimistic. Therefore, in the present study, resubstitution was employed exclusively as an exploratory tool to compare feature selection strategies, assess algorithm learning behavior, and identify stable predictors of mortality.

All final performance evaluations were based on independent validation procedures, including cross-validation and testing on a completely unseen independent dataset. The independent test set was excluded from feature selection, model training, algorithm comparison, and parameter optimization processes, thereby preventing information leakage and ensuring an unbiased assessment of model generalizability.

Overall, the use of multiple validation strategies increased the transparency of the analysis framework and strengthened the methodological rigor of the study. The consistent identification of the same core predictors across multiple validation and feature selection strategies strengthens the robustness of the proposed framework and supports their potential clinical relevance for ICU mortality prediction.

## 4. Results

### 4.1. Experiment Set-1: Clinically Interpretable Feature Set

In this experiment, mortality prediction was performed using 12 radiographic and demographic features. Initial analyses identified the Bagged Trees classifier as the best-performing model using the complete 12-feature set. Subsequently, feature reduction was performed using the MRMR and Permutation Importance methods, and four core features (Cobb angle, age, bilateral pleural effusion, and unilateral infiltration) were consistently selected by both approaches.

Despite reducing the number of features from 12 to 4, no substantial decrease in predictive performance was observed. These results indicate that a substantial reduction in feature dimensionality can be achieved without compromising predictive performance. In particular, the Subspace KNN classifier achieved the highest discriminative performance when the four core features were used. Analyses performed using feature subsets obtained from the MRMR and Permutation Importance methods further demonstrated that different feature selection strategies may influence model behavior; however, reliable mortality prediction can still be achieved using a limited number of clinically interpretable features. These findings demonstrate that appropriate feature selection can substantially reduce model complexity while preserving competitive predictive performance, thereby improving the interpretability and potential clinical relevance of the proposed models.

### 4.2. Experiment Set-2: Comparative Evaluation of Feature Selection Strategies

In the second experiment, 74 radiographic, radiological, and image-derived features were evaluated to compare different validation and feature selection strategies. Initial analyses identified the Coarse KNN algorithm as the best-performing classifier under the complete 74-feature configuration; therefore, it served as the reference model for subsequent feature selection analyses.

Model robustness was assessed using multiple validation approaches, including cross-validation, hold-out validation, and resubstitution analysis. Resubstitution was not employed as a final performance evaluation technique but rather as an exploratory tool for comparing feature selection methods and investigating algorithm behavior.

Comparative analyses using ANOVA, Chi-Square, Kruskal–Wallis, MRMR, ReliefF, and Shapley-based feature selection methods revealed that Cobb angle, bilateral infiltrates, bilateral pleural effusion, and unilateral pleural effusion were consistently selected across all approaches. This finding suggests that these variables represent stable and clinically interpretable predictors of ICU mortality. The detailed ranking of the top features identified by each feature selection method is presented in Supplementary Table S3.

Among the evaluated feature selection strategies, MRMR and Kruskal–Wallis produced the highest-performing feature subsets, while the remaining methods yielded comparable results. Furthermore, the common four-feature core set preserved a substantial proportion of the predictive performance achieved by the full feature set, despite the considerable reduction in dimensionality.

### 4.3. Overall Findings

When the results from both experimental datasets were considered together, it was observed that ICU mortality could be predicted successfully using either a limited number of clinically interpretable features or a larger high-dimensional feature space derived from chest radiographs.

The findings further indicate that feature selection strategies play an important role in identifying stable and informative predictors while reducing model complexity. In particular, variables that were consistently selected across multiple feature selection methods demonstrated strong predictive value and may represent clinically relevant indicators of patient outcomes.

Overall, the findings demonstrate that clinically interpretable radiographic and demographic features, when combined with appropriate feature selection strategies, enable the development of accurate, explainable, and computationally efficient mortality prediction models. These findings provide a practical foundation for the development of transparent, clinically interpretable, and computationally efficient decision-support systems for early ICU mortality risk stratification.

## 5. Discussion

### 5.1. Principal Findings

The present study investigated the influence of feature type, feature dimensionality, and feature selection strategies on intensive care unit (ICU) mortality prediction using chest radiographs and demographic information. Unlike many previous studies that primarily focused on maximizing predictive performance through increasingly complex machine learning models, the present work aimed to identify a compact, stable, and clinically interpretable set of predictors while maintaining high classification performance. Two complementary experimental frameworks were therefore evaluated: a clinically interpretable dataset composed of radiographic and demographic variables, and a higher-dimensional dataset incorporating radiographic, radiomic, and image-derived features.

Across both experimental frameworks, a consistent finding emerged that substantial reductions in feature dimensionality could be achieved without meaningful deterioration in predictive performance. Although the second experimental dataset initially contained 74 candidate variables, multiple independent feature selection techniques consistently converged on a small group of highly informative predictors. Similarly, in the clinically interpretable dataset, reducing the predictor set from 12 to four variables resulted in only minimal changes in classification performance. Collectively, these findings suggest that careful feature selection may contribute to model performance as much as, or even more than, the selection of the machine learning algorithm itself. This observation highlights the importance of identifying robust and biologically meaningful predictors rather than relying solely on increasingly complex classification models.

### 5.2. Clinical Interpretation of the Selected Predictors

The consistent identification of several predictors across multiple feature selection methods provides additional confidence that these variables contain clinically relevant information rather than representing method-specific artifacts. Among the selected variables, bilateral infiltrates, bilateral pleural effusion, unilateral pleural effusion, and age have all been previously associated with adverse clinical outcomes in critically ill patients. Bilateral pulmonary infiltrates frequently reflect diffuse pulmonary involvement caused by pneumonia, pulmonary edema, acute respiratory distress syndrome (ARDS), or widespread inflammatory processes, all of which are well-recognized contributors to respiratory failure and increased mortality. Similarly, pleural effusion may indicate underlying cardiac dysfunction, renal impairment, fluid overload, infection, or severe systemic disease, making it a clinically meaningful marker of disease severity rather than an isolated radiographic finding.

Age also emerged as one of the most influential predictors within the clinically interpretable feature set. This finding is consistent with previous studies demonstrating that advanced age is associated with reduced physiological reserve, impaired immune function, increased frailty, and a higher prevalence of chronic comorbidities, all of which contribute to poorer outcomes among ICU patients. The persistent selection of age across the feature reduction process therefore supports the biological plausibility of the proposed prediction model.

Particular attention should be given to the repeated selection of the Cobb angle, as this parameter is conventionally used for the assessment of spinal deformities, particularly scoliosis. In the present study, however, the Cobb angle should not be interpreted as a diagnostic marker of scoliosis. Instead, it represents an image-derived quantitative descriptor reflecting thoracic spinal alignment and overall thoracic morphology visible on chest radiographs. Variations in thoracic alignment may indirectly capture age-related postural alterations, chronic musculoskeletal changes, thoracic deformity, reduced chest wall compliance, or generalized frailty, all of which may influence respiratory mechanics and physiological reserve in critically ill patients. Consequently, the association observed in this study is more likely to reflect the combined effects of underlying structural and physiological conditions rather than the presence of scoliosis itself.

Nevertheless, the biological mechanisms underlying this association should be interpreted cautiously. Because this study was retrospective and observational, the identified relationship does not imply causality. It is also possible that the predictive value of the Cobb angle partially reflects unmeasured confounding factors, including underlying chronic diseases, differences in patient positioning during image acquisition, variations in thoracic anatomy, or other clinical characteristics that were not incorporated into the present dataset. Future multicenter studies integrating comprehensive clinical variables together with longitudinal imaging data will be necessary to determine whether the Cobb angle represents an independent prognostic marker or serves as a surrogate indicator of overall patient frailty and disease severity.

It should also be recognized that Cobb angle measurements obtained from chest radiographs may be influenced by several technical and patient-related factors, including patient age, body positioning during image acquisition, image rotation, and pre-existing chronic spinal deformities. Although all radiographs in the present study were acquired using a standardized imaging protocol and processed through the same measurement pipeline, these factors cannot be completely eliminated in retrospective clinical datasets. Therefore, the predictive value of the Cobb angle observed in this study should not be interpreted as evidence of a direct causal relationship with ICU mortality. Rather, it may represent a surrogate imaging biomarker reflecting the combined effects of thoracic morphology, frailty, chronic musculoskeletal changes, and overall physiological reserve. Future multicenter studies incorporating standardized image acquisition protocols and additional clinical covariates are warranted to further investigate the independent prognostic significance of the Cobb angle.

### 5.3. Comparison with Previous Literature

The findings of the present study are generally consistent with the growing body of literature demonstrating the effectiveness of machine learning approaches for mortality prediction and clinical risk stratification. Previous studies have shown that integrating imaging, clinical, laboratory, or demographic variables with appropriate machine learning algorithms can substantially improve predictive performance. However, many published models rely on large numbers of heterogeneous predictors, complex feature engineering procedures, or multimodal datasets, which may reduce model interpretability and limit their practical implementation in routine clinical settings.

Unlike many previous investigations, the primary objective of the present study was not simply to maximize classification accuracy but to identify a stable and clinically interpretable feature set capable of maintaining high predictive performance after substantial dimensionality reduction. The observation that only a limited number of radiographic and demographic variables preserved most of the predictive capability suggests that clinically meaningful information may be concentrated within a relatively small subset of carefully selected features.

The comparative analysis summarized in Table 9 further illustrates these methodological differences. While previous studies have employed various feature selection techniques, including ReliefF, SHAP, recursive feature elimination (RFE), Pearson correlation analysis, and gradient boosting–based importance measures, the present study systematically compared multiple statistical and machine learning-based feature selection approaches within the same patient cohort. The consistent identification of several common predictors across six independent feature selection methods provides additional evidence regarding the robustness and stability of the selected variables.

Another distinguishing aspect of the present study is its emphasis on explainability. As interest in explainable artificial intelligence continues to grow in medical applications, models based on a limited number of clinically interpretable variables may offer important advantages over highly complex black-box models. Such models are easier to validate, interpret, and potentially integrate into routine clinical workflows, thereby facilitating clinician acceptance and supporting transparent decision-making in critical care environments.

Table 9 summarizes methodological comparison of previous machine learning studies and the present study. The table summarizes differences in sample size, data type, feature dimensionality, feature selection strategies, machine learning algorithms, and predictive performance. Compared with previous studies, the present study achieved competitive performance using a relatively small number of clinically interpretable radiographic and demographic features.

### 5.4. Clinical Implications

Beyond its predictive performance, the proposed framework has several practical implications for clinical decision-making in the intensive care unit. Early identification of patients at increased risk of mortality remains a major challenge in critical care medicine, where timely intervention can significantly influence patient outcomes. Because chest radiography is routinely performed during ICU admission, the use of radiographic and demographic features for automated risk prediction has the potential to provide clinically relevant information without requiring additional diagnostic procedures or increasing healthcare costs.

An important advantage of the proposed approach is its reliance on a relatively small number of clinically interpretable variables. Unlike many artificial intelligence models that depend on hundreds or thousands of complex imaging features, the present model demonstrates that competitive predictive performance can be achieved using a limited set of understandable radiographic findings together with basic demographic information. This level of interpretability is particularly important in medical applications, where clinicians are more likely to trust and adopt decision-support systems whose predictions can be explained using familiar clinical concepts.

The results also emphasize the importance of feature selection as an integral component of medical artificial intelligence rather than merely a data preprocessing step. The observation that similar predictive performance was maintained after substantial dimensionality reduction suggests that carefully selected variables may improve not only computational efficiency but also model robustness and generalizability. Consequently, future clinical decision-support systems may benefit from prioritizing feature stability and interpretability in addition to maximizing predictive accuracy.

Although the proposed model is not intended to replace physician judgment, it may serve as an adjunctive tool for early risk stratification. Patients identified as being at higher risk could be prioritized for closer monitoring, earlier diagnostic evaluation, or more intensive therapeutic interventions. Such applications are particularly relevant in high-volume intensive care units, where rapid and objective assessment of patient severity is essential for optimizing resource allocation and clinical workflow.

### 5.5. Strengths of the Study

The present study has several strengths that distinguish it from previous investigations. First, instead of focusing exclusively on maximizing predictive accuracy, the study systematically examined the combined effects of feature type, feature dimensionality, and feature selection strategy on ICU mortality prediction. This comprehensive evaluation provides a broader understanding of how different methodological choices influence model performance and interpretability.

Second, two complementary experimental frameworks were investigated. The first emphasized clinically interpretable radiographic and demographic variables, whereas the second incorporated a larger and more diverse feature space containing radiographic, radiomic, and image-derived characteristics. Evaluating both approaches within the same study enabled a direct comparison between model simplicity and predictive performance, thereby providing practical insights for future clinical applications.

Another important strength is the systematic comparison of six independent feature selection methods, including ANOVA, Chi-Square, Kruskal–Wallis, MRMR, ReliefF, and SHAP. Rather than relying on a single feature selection algorithm, the present study identified predictors that were consistently selected across multiple independent approaches. This strategy increases confidence that the selected variables represent robust and reproducible predictors instead of artifacts associated with a particular statistical technique.

Finally, the emphasis placed on model interpretability represents another important contribution of the present work. In contrast to many recently proposed black-box artificial intelligence models, the present framework demonstrates that competitive predictive performance can be achieved while maintaining transparency and clinical interpretability. Such explainable models are more likely to gain acceptance among healthcare professionals and may facilitate the future integration of artificial intelligence into routine intensive care practice.

Although the proposed approach demonstrated encouraging discrimination performance in the independent test set, external validation and formal statistical comparisons are required before drawing conclusions regarding its generalizability or clinical implementation.

### 5.6. Limitations and Future Directions

Despite the encouraging findings, several limitations should be considered when interpreting the results of the present study.

First, the analyses were conducted using a retrospective dataset collected from a single-center intensive care unit, which may limit the generalizability of the findings to other institutions, patient populations, and clinical settings. Although an independent test set was reserved for final evaluation and multiple validation strategies, including cross-validation and hold-out validation, were employed to assess model robustness, the independent test cohort consisted of only 100 patients. Furthermore, external validation using datasets from other institutions was not performed. Consequently, the reported performance estimates should be interpreted with appropriate caution until they are confirmed in larger, multicenter cohorts.

Second, all radiographic measurements and image-derived features were extracted from first-day portable frontal chest radiograph obtained at ICU admission. Therefore, temporal changes in radiographic findings, disease progression, and longitudinal imaging assessments were not evaluated. Future studies incorporating serial chest radiographs may provide additional prognostic information and improve the temporal assessment of disease evolution.

In addition, the present study focused primarily on discrimination performance using an independent test dataset. Calibration analysis (e.g., calibration plots and Brier score), decision-threshold optimization, and decision-curve analysis were beyond the scope of the current study. These complementary evaluations should be incorporated in future studies involving larger multicenter cohorts and external validation to further establish the clinical applicability of the proposed prediction model.

Another limitation is that several radiographic variables, including unilateral and bilateral infiltrates, pleural effusions, pneumothorax, calcified aorta, and cardiomegaly, were determined through expert radiologist assessment. Consequently, observer-dependent variability and inter-observer variability cannot be completely excluded. Future investigations involving multiple radiologists and formal inter-rater agreement analyses would further improve the reproducibility and robustness of the proposed methodology.

Furthermore, the present study intentionally focused on radiographic and demographic variables to evaluate the isolated contribution of chest radiographic features and feature selection strategies. Comprehensive clinical information, including admission diagnoses, comorbidities, laboratory findings, physiological parameters, and severity scores such as APACHE II and SOFA, was not available in the retrospective imaging dataset. The integration of these complementary clinical variables with radiographic features may further improve predictive performance and should be explored in future studies.

As the study was based on routine ICU chest radiographs retrieved from the hospital PACS archive, variations in acquisition geometry may have existed among patients. Although all image-derived measurements were obtained using a standardized automated processing pipeline, projection-related variability may have influenced projection-dependent parameters such as the cardiothoracic ratio.

In addition, because of the retrospective observational design, residual confounding cannot be completely excluded. Unmeasured factors, including differences in treatment strategies, medication use, ventilatory support, underlying chronic diseases, patient positioning during image acquisition, and other clinical characteristics, may have influenced both the radiographic findings and mortality outcomes. Therefore, the observed associations should be interpreted as predictive rather than causal.

Finally, although the Cobb angle was consistently identified as one of the most stable predictors across multiple feature selection methods, its biological relationship with ICU mortality remains incompletely understood. In the present study, the Cobb angle was interpreted as an image-derived descriptor of thoracic morphology rather than a diagnostic indicator of scoliosis. Nevertheless, whether this variable represents an independent prognostic marker or serves as a surrogate for age-related structural alterations, frailty, or other unmeasured clinical factors requires further investigation in larger prospective multicenter studies.

Despite these limitations, the present study provides a comprehensive and systematic comparison of multiple feature selection strategies and demonstrates that accurate, interpretable, and computationally efficient ICU mortality prediction models can be developed using a limited number of clinically meaningful radiographic and demographic variables. In addition, the findings should be interpreted considering the relatively limited sample size and the absence of external validation, which may affect the generalizability of the proposed framework. We anticipate that these findings will provide a useful foundation for future multicenter studies and contribute to the development of clinically applicable and explainable artificial intelligence–based decision-support systems for intensive care medicine.

## 6. Conclusions

This study investigated the influence of feature type, feature dimensionality, and feature selection strategies on ICU mortality prediction using chest radiographic and demographic data. Two complementary experimental frameworks were evaluated: a clinically interpretable feature set consisting of radiographic and demographic variables, and a higher-dimensional feature space incorporating radiographic, radiomic, and image-derived features.

The findings demonstrate that accurate mortality prediction can be achieved using a limited number of carefully selected features. In both experimental frameworks, substantial feature reduction decreased model complexity while preserving competitive predictive performance. Furthermore, several predictors, including Cobb angle, bilateral infiltrates, bilateral pleural effusion, and unilateral pleural effusion, were consistently identified across multiple feature selection methods, supporting their robustness as candidate predictors of ICU mortality.

The study further highlights that appropriate feature selection may contribute to model performance as much as the choice of the machine learning algorithm itself. These findings emphasize the value of identifying stable and clinically interpretable predictors rather than relying exclusively on increasingly complex high-dimensional models. Consequently, the proposed framework provides a systematic approach for developing transparent, explainable, and computationally efficient machine learning models for mortality prediction.

Nevertheless, the present findings should be interpreted in light of the study’s retrospective single-center design and the absence of external validation. Therefore, although the proposed approach demonstrated promising predictive performance, prospective multicenter validation using larger and more diverse patient populations will be essential before its potential clinical implementation can be established.

Overall, this study provides new insights into the role of feature selection in ICU mortality prediction and offers a foundation for future research aimed at developing robust, explainable, and clinically deployable artificial intelligence–based decision-support systems for critical care.

## Figures and Tables

**Table 1:** Baseline Characteristics of the Study Population.

Variable	Survivors	Non-Survivors
***n***	258	242
**Age (mean ± SD)**	70.6 ± 16.4	75.0 ± 12.5
**Age range**	20–97	37–109
**Male**	139 (53.9%)	126 (52.1%)
**Female**	119 (46.1%)	116 (47.9%)

**Table 2:** Comparative performance of the three best-performing machine learning algorithms using the complete 12-feature set.

Algorithm	AUC	Sensitivity (%)	Specificity (%)	Accuracy (%)	F1-Score (%)
**Bagged Trees**	0.95	0.76	1.00	0.88	0.86
**Boosted Trees**	0.92	0.65	0.98	0.82	0.81
**RUSBoosted Trees**	0.87	0.67	0.88	0.78	0.78

**Table 3:** Comparative performance of the three best-performing machine learning algorithms using the four-feature subset selected by the MRMR algorithm.

Algorithm	AUC	Sensitivity (%)	Specificity (%)	Accuracy (%)	F1-Score (%)
**Bagged Tree**	0.88	0.65	1.00	0.83	0.79
**Fine Tree**	0.82	0.78	0.96	0.78	0.77
**RUSBoosted Tree**	0.81	0.77	0.78	0.77	0.77

**Table 4:** Comparative performance of the three best-performing machine learning algorithms using the four core features selected jointly by the MRMR and Permutation Importance methods.

Algorithm	AUC	Sensitivity (%)	Specificity (%)	Accuracy (%)	F1-Score (%)
**Subspace KNN**	0.96	0.63	1.00	0.82	0.78
**Bagged Trees**	0.88	0.61	1.00	0.81	0.80
**Boosted Trees**	0.93	0.55	0.98	0.77	0.76

**Table 5:** Comparative performance of the three best-performing machine learning algorithms using the four features selected by the Permutation Importance method.

Algorithm	AUC	Sensitivity (%)	Specificity (%)	Accuracy (%)	F1-Score (%)
**Weighted KNN**	0.98	0.55	1.00	0.78	0.71
**Bagged Tree**	0.93	0.82	0.82	0.82	0.82
**RUSBoosted Tree**	0.90	0.82	0.82	0.82	0.82

**Table 6:** Comparative performance of the three best-performing machine learning algorithms using the complete 74-feature dataset under cross-validation.

Algorithm	AUC	Sensitivity (%)	Specificity (%)	Accuracy (%)	F1-Score (%)
**Coarse KNN**	0.77	0.67	0.82	0.75	0.73
**Binary GLM**	0.75	0.70	0.74	0.73	0.70
**Linear Discriminant**	0.73	0.72	0.72	0.72	0.71

**Table 7:** Comparative performance of the three best-performing machine learning algorithms using the complete 74-feature dataset under hold-out validation.

Algorithm	AUC	Sensitivity (%)	Specificity (%)	Accuracy (%)	F1-Score (%)
**Coarse KNN**	0.769	0.77	0.72	0.76	0.76
**Binary GLM**	0.749	0.70	0.74	0.73	0.70
**Linear Discriminant**	0.731	0.72	0.72	0.72	0.71

**Table 8:** Frequency of feature selection across six feature selection methods.

Feature	Number of Methods Selecting the Feature	Methods
Cobb Angle	6/6	ANOVA, Chi-Square, Kruskal–Wallis, MRMR, ReliefF, Shapley
Bilateral Infiltrates	6/6	ANOVA, Chi-Square, Kruskal–Wallis, MRMR, ReliefF, Shapley
Bilateral Pleural Effusion	6/6	ANOVA, Chi-Square, Kruskal–Wallis, MRMR, ReliefF, Shapley
Unilateral Pleural Effusion	6/6	ANOVA, Chi-Square, Kruskal–Wallis, MRMR, ReliefF, Shapley
CTR	3/6	Chi-Square, MRMR, Shapley
Pneumothorax	4/6	Chi-Square, Kruskal–Wallis, MRMR, ReliefF
Haralick_13_90	4/6	ANOVA, Kruskal–Wallis, MRMR, Shapley
Image Entropy	3/6	Chi-Square, Kruskal–Wallis, Shapley

**Table 9:** Studies reported in the literature.

Study	Sample Size	Data Type	Initial Number of Features	Feature Selection Method	Machine Learning Algorithm	Best AUC
Gok et al. (2025) [13]	510 patients	Portable frontal chest radiograph	74 radiomic features	ReliefF	Subspace KNN	0.88
Genç et al. (2025) [14]	220 patients	Clinical, laboratory, and scoring data	85 features	SHAP	CatBoost	0.90
Dou et al. (2024) [10]	UCI Heart Disease Dataset	Clinical data	13 features	Pearson Correlation, Chi-Square, RFE, LightGBM	Ensemble Stacking	0.95
Ma et al. (2020) [26]	633 subjects	Clinical and genetic data (101 SNPs + 5 clinical variables)	106 features	XGBoost Feature Importance	XGBoost	0.94
Vairale and Dubey (2025) [21]	2149 records	Demographic and clinical data	35 features	Feature selection applied	XGBoost	0.95
Present Study	500 patients	Portable frontal chest radiograph and demographic data	12 and 74 features (two experimental datasets)	MRMR and Permutation Importance	Weighted KNN	0.96

## Data Availability

The data used in this study were obtained from the Sakarya University Training and Research Hospital database. Due to ethical and privacy restrictions, these data are not publicly available but can be provided upon reasonable request from the corresponding author, in compliance with institutional policies.

## Supplementary Materials

The following supporting information can be downloaded at: https://www.mdpi.com/article/10.3390/diagnostics16152391/s1, Table S1: Example radiomic and texture feature set; Table S2: Complete list of all Haralick and GLCM texture features extracted in this study; Table S3: Comparative ranking of the top 15 features identified by the six feature selection methods (ANOVA, Chi-Square, Kruskal–Wallis, MRMR, ReliefF, and SHAP).

## References

[1] Lau V.I., Hammal F., Sligl W.I., Karvellas C.J., Kutsogiannis D.J., Zampieri F.G., Wilcox M.E., Fiest K.M., Niven D.J., Stelfox H.T. (2025). Intensive care unit and hospital mortality for non-COVID critically ill patients before, and during the COVID-19 pandemic in Alberta hospitals: Retrospective, observational cohort study. medRxiv.

[2] Sayar B., Özyılmaz C., Dindar E.K., Kuşlu S., Taş Çiftçibaşı F. (2025). Assessment of mortality in intensive care: A critical look at risk factors and care practices. J. Orig. Stud..

[3] Kohn R., Weissman G.E., Wang W., Ingraham N.E., Scott S., Bayes B., Anesi G.L., Halpern S.D., Kipnis P., Liu V.X. (2023). Prediction of in-hospital mortality among intensive care unit patients using modified daily Laboratory-based Acute Physiology Scores, version 2 (LAPS2). Med. Care.

[4] Broder J., Broder J. (2011). Imaging the chest: The chest radiograph. Diagnostic Imaging for the Emergency Physician.

[5] Bagnera S., Bisanti F., Tibaldi C., Pasquino M., Berrino G., Ferraro R., Patania S. (2020). Performance of radiologists in the evaluation of the chest radiography with the use of a “new software score” in coronavirus disease 2019 pneumonia suspected patients. J. Clin. Imaging Sci..

[6] Matusik P.S., Popiela T.J., Matusik P.T. (2025). Novel Indexes in the Assessment of Cardiac Enlargement Using Chest Radiography: A New Look at an Old Problem. J. Clin. Med..

[7] Polis B., Zawadzka-Fabijan A., Fabijan R., Kosińska R., Nowostawska E., Fabijan A. (2025). Exploring BiomedCLIP’s capabilities in medical image analysis: A focus on scoliosis detection and severity assessment. Appl. Sci..

[8] Fabijan A., Fabijan R., Zawadzka-Fabijan A., Nowosławska E., Zakrzewski K., Polis B. (2023). Evaluating scoliosis severity based on posturographic X-ray images using a contrastive language–image pretraining model. Diagnostics.

[9] Suri A., Tang S., Kargilis D., Taratuta E., Kneeland B.J., Choi G., Agarwal A., Anabarayone N., Xu W., Parente J.B. (2023). Conquering the Cobb angle: A deep learning algorithm for automated, hardware-invariant measurement of Cobb angle on radiographs in patients with scoliosis. Radiol. Artif. Intell..

[10] Dou Y., Liu J., Meng W., Zhang Y. (2024). Comparative analysis of supervised learning algorithms for prediction of cardiovascular diseases. Technol. Health Care.

[11] García M.V., Bouza-Rodríguez J.-B., Comesaña-Campos A. (2025). Convolutional Neural Network-Based Approach for Cobb Angle Measurement Using Mask R-CNN. Diagnostics.

[12] Zhu T., Xu K., Son W., Linton-Reid K., Boubnovski-Martell M., Grech-Sollars M., Lain A.D., Posma J.M. (2025). Designing a computer-assisted diagnosis system for cardiomegaly detection and radiology report generation. PLoS Digit. Health.

[13] Gok O., Cavus T.F., Genc A.C., Yaylaci S., Ayhan L.T. (2025). Mortality prediction from patient’s first day PAAC radiograph in internal medicine intensive care unit using artificial intelligence methods. Diagnostics.

[14] Genç A.C., Özmen E., Çekiç D., İşsever K., Türkoğlu Genç F., Genç A.B., Toçoğlu A., Durmaz Y., Özkök H., Yaylacı S. (2025). Comprehensive analyses: Using machine learning models for mortality prediction in the intensive care unit of internal medicine. J. Investig. Med..

[15] Torres F.S., Eifer D.A., Tijmes F.S., Nguyen E.T., Hanneman K. (2021). Diagnostic performance of chest radiography measurements for the assessment of cardiac chamber enlargement. Can. Med. Assoc. J..

[16] Vrtovec T., Pernuš F., Likar B. (2009). A review of methods for quantitative evaluation of spinal curvature. Eur. Spine J..

[17] Liu D., Zhang L., Yang J., Lin A. (2023). Lenke Classification of Scoliosis Based on Segmentation Network and Adaptive Shape Descriptor. Appl. Sci..

[18] Simkus P., Gutierrez Gimeno M., Banisauskaite A., Neththipola W., Nair J., Al-Mukhtar M. (2021). Limitations of cardiothoracic ratio derived from chest radiographs to predict real heart size: Comparison with magnetic resonance imaging. Insights Into Imaging.

[19] Aykanat M., Kılıç Ö., Kurt B., Saryal S. (2020). Lung Disease Classification Using Machine Learning Algorithms. Int. J. Appl. Math. Electron. Comput..

[20] (2022). Performance analysis of machine learning algorithms in predicting pulmonary diseases. 2022 International Conference on Machine Learning, Big Data, Cloud and Parallel Computing (COM-IT-CON).

[21] Vairale P., Dubey V. A comprehensive comparison of machine learning classification techniques for early detection of Alzheimer’s disorder. Proceedings of the 2025 4th International Conference on Computing, Communication, and Intelligent Systems (ICCCIT).

[22] Brakohiapa E.K.K., Botwe B.O., Sarkodie B.D., Ofori E.K., Coleman J. (2017). Radiographic determination of cardiomegaly using cardiothoracic ratio and transverse cardiac diameter: Can one size fit all? Part one. Pan Afr. Med. J..

[23] Slade R., Alikhan R., Wise M.P., Germain L., Stanworth S., Morgan M. (2019). Impact of blood group on survival following critical illness: A single-centre retrospective observational study. BMJ Open Respir. Res..

[24] Kander T., Bjurström M.F., Frigyesi A., Jöud M., Nilsson C.U. (2022). ABO and RhD blood group are not associated with mortality and morbidity in critically ill patients; a multicentre observational study of 29 512 patients. BMC Anesthesiol..

[25] Reddy I.S., Shaik A.P. (2025). Abo Blood Types and Mortality Following Critical Illness: A Single Centre Retrospective Observational Study. Indian J. Crit. Care Med. Peer-Rev. Off. Publ. Indian Soc. Crit. Care Med..

[26] Ma X., Wu Y., Zhang L., Yuan W., Yan L., Fan S., Lian Y., Zhu X., Gao J., Zhao J. (2020). Comparison and development of machine learning tools for the prediction of chronic obstructive pulmonary disease in the Chinese population. J. Transl. Med..

