# Supplementary material for: Comparative Evaluation of Feature Selection Strategies for ICU Mortality Prediction Using Chest Radiographic, Radiomic, and Demographic Features
Source: Diagnostics (Basel). 2026 Jul 30;16(15):2391. doi: 10.3390/diagnostics16152391 (PMC13465637; doi:10.3390/diagnostics16152391)
Supplement: Supplementary file 1 [file diagnostics-16-02391-s001.zip › TableS2.pdf]

Supplementary Table S2. Complete list of all Haralick and GLCM texture features extracted in this study.

Cardiomegaly (CTR > 1)	GLCM Contrast	Haralick_11_0	Haralick_1_90	Haralick_4_135
Infiltration (unilateral)	GLCM Homogeneity	Haralick_12_0	Haralick_2_90	Haralick_5_135
Infiltration (bilateral)	GLCM Energy	Haralick_13_0	Haralick_3_90	Haralick_6_135
Pleural effusion (unilateral)	GLCM Corr.	Haralick_1_45	Haralick_4_90	Haralick_7_135
Pleural effusion (bilateral)	GLCM Diss.	Haralick_2_45	Haralick_5_90	Haralick_8_135
Pneumothorax	GLCM ASM	Haralick_3_45	Haralick_6_90	Haralick_9_135
Calcified aorta	Haralick_1_0	Haralick_4_45	Haralick_7_90	Haralick_10_135
Cardiothoracic Ratio (CTR)	Haralick_2_0	Haralick_5_45	Haralick_8_90	Haralick_11_135
Cobb Angle	Haralick_3_0	Haralick_6_45	Haralick_9_90	Haralick_12_135
Mean Intensity	Haralick_4_0	Haralick_7_45	Haralick_15_90	Haralick_13_135
Variance	Haralick_5_0	Haralick_8_45	Haralick_11_90	
Skewness	Haralick_6_0	Haralick_9_45	Haralick_12_90	
Kurtosis	Haralick_7_0	Haralick_15_45	Haralick_13_90	
Median Intensity	Haralick_8_0	Haralick_11_45	Haralick_1_135	
Intensity Range	Haralick_9_0	Haralick_12_45	Haralick_2_135	
Image Entropy	Haralick_15_0	Haralick_13_45	Haralick_3_135	

The 74 features listed in the table above were used in the performance analyses.
