# Supplementary material for: Comparative Evaluation of Feature Selection Strategies for ICU Mortality Prediction Using Chest Radiographic, Radiomic, and Demographic Features
Source: Diagnostics (Basel). 2026 Jul 30;16(15):2391. doi: 10.3390/diagnostics16152391 (PMC13465637; doi:10.3390/diagnostics16152391)
Supplement: Supplementary file 1 [file diagnostics-16-02391-s001.zip › TableS3.pdf]

Supplementary Table S3. Comparative ranking of the top 15 features identified by the six feature selection methods (ANOVA, Chi-Square, Kruskal–Wallis, MRMR, ReliefF, and SHAP).

Feature	ANOVA	Chi-Square	Kruskal–Wallis	MRMR	ReliefF	SHAP
Cobb Angle	1	1	1	1	4	1
Bilateral Infiltrates	2	2	2	3	1	4
Bilateral Pleural Effusion	3	4	3	7	2	3
Unilateral Pleural Effusion	4	5	4	2	3	2

Supplementary Table S3. Comparative ranking of the top 15 features identified by the six feature selection methods (ANOVA, Chi-Square, Kruskal–Wallis, MRMR, ReliefF, and SHAP). Features consistently ranked among the highest across multiple methods were considered the core predictive feature set used in subsequent analyses.
